# Growing Season Lengthens in a North American Deciduous Woody Community From 1993 to 2021

**DOI:** 10.1002/ece3.71226

**Published:** 2025-05-19

**Authors:** Carol K. Augspurger, David N. Zaya

**Affiliations:** ^1^ Department of Plant Biology University of Illinois Urbana Illinois USA; ^2^ Illinois Natural History Survey University of Illinois Champaign Illinois USA

**Keywords:** autumn phenology, climate change, long‐term, phenology, spring phenology

## Abstract

1. Observations of both spring and autumn phenological events were made annually over 29 years (1993–2021) for 22 taxa of multiple growth forms in a mature deciduous forest remnant near Urbana, Illinois, USA. Temporal trends in event dates, trends in stage durations, and associations with weather variables were analyzed with linear mixed‐effect models. Species were grouped together in analyses based on seasonality.
2. Spring event dates for most species advanced from 1.2 to 3.0 days/decade, while durations of spring stages shortened from 0.3 to 0.6 days/decade.
3. Autumn event dates for most species delayed from 1.2 to 3.3 days/decade, while durations of autumn stages lengthened from 0.8 to 3.8 days/decade.
4. Overall, the duration of the growing season lengthened for 88% of species (mean of 4.7 days/decade), with greater delays in autumn phenology for canopy trees and greater advances in spring phenology for other woody life forms.
5. In spring, warmer mean daily temperatures were associated with advances in dates of phenological events. In autumn, minimum daily temperature in the preceding month(s) had the highest predictive power for seasonal groups, except those with *Aesculus glabra* .
6. In autumn, most species had both a delay in phenology and a strong weather predictor, minimum daily temperature in September, that increased significantly through 29 years. In spring, some concordance between advancing event dates and warming spring temperatures were evident after removing data from 2018 to 2021 with especially high variability in spring temperatures.
7. This study supports the hypothesis that climate change is showing a pronounced association with a delay in autumn leaf coloration, and less so an advance of spring leaf expansion. These changes can affect ecological processes, including plant productivity and carbon uptake/storage, assembly of communities, interactions between trophic levels, and species ranges and invasions.

## Graphical abstract

For a North American deciduous woody community, the study demonstrates that, over 29 years, increased phenological advances in spring and delays in autumn have led to a growing season lengthening of 4.7 days/decade. Warming of spring mean daily temperatures and autumn minimum temperatures predicted these phenological changes, thus supporting the hypothesis that climate change is associated with these changes in phenology. These changes can affect ecological processes, including plant productivity and carbon uptake/storage, assembly of communities, interactions between tropic levels, and species ranges and invasions.

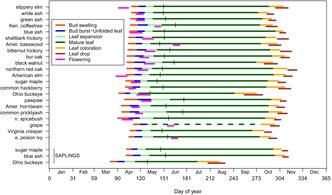

## 1 Introduction

Phenological patterns of woody plant species, including bud burst and leaf expansion in spring and leaf coloration and leaf drop in autumn, have been changing in seasonal temperate deciduous forests (Menzel 2000; Ge et al. 2015). Many studies show that rising spring temperatures predict advances in the timing of spring events (Menzel et al. 2006; Piao et al. 2019), with some exceptions and complications (Roberts et al. 2015). Fewer studies of autumn events show a delay in timing with warmer autumn temperatures (Gill et al. 2015; Gallinat et al. 2015). These altered leaf stages result in longer growing seasons (Jeong et al. 2011), and may impact ecological processes, including community assembly and species niches (Ponti and Sannolo 2023), community interactions within and between trophic levels (Nakazawa and Doi 2011; Renner and Zohner 2018; Belitz et al. 2025), species' ranges (Morin et al. 2007), and invasions (Polgar et al. 2014). Additionally, a longer growing season potentially brings longer periods of photosynthesis and carbon uptake and storage by canopy trees (Keenan et al. 2014), although the results are mixed as to whether this potential is realized (Dunn et al. 2007; Piao et al. 2008; Wu et al. 2013).

A longer growing season by canopy trees may increase the period of shade experienced by understory species and saplings dependent on high light before canopy closure and/or after canopy opening (Renner and Zohner 2018; Augspurger and Salk 2024). However, the focus of phenological studies of woody species has been on canopy trees, and not the growth forms of saplings, vines, treelets, and shrubs (but see Ge et al. 2015).

The amount of autumn delay, and, by extension, the amount of lengthening of the growing season, can be constrained by increases in spring and summer productivity (Zani et al. 2020). Additionally, Zohner et al. (2023) report that warm and cold years differ in their effect on autumn phenology. In cold years, slow spring development results in a late beginning and rapid movement toward an early ending of senescence; conversely, in warm years, rapid spring development causes early beginning and slow movement to a later ending of senescence.

Europe (Wang et al. 2016; Wesołowski and Rowiński 2006) and parts of Asia (Ge et al. 2015) have long‐term observational studies of the phenology of their deciduous forests for a large number of species, years, and countries. Long‐term North America studies were focused historically on northeastern USA (Harvard Forest: O'Keefe 2024; Hubbard Brook Ecosystem Study 2022) and midwestern USA (Morin et al. 2009; Yu et al. 2016; Donnelly et al. 2017). The USA National Phenology Network is increasingly being used as its data set becomes longer and more widespread (Crimmins et al. 2022). In North America, it remains unclear how climate change in recent decades is affecting the growing season of mature deciduous forests and whether any changes are associated with spring or autumn phenology.

Temperature and precipitation are key factors that predict phenology of deciduous trees. In spring, accumulating warmth from rising temperatures forces leaf development (Polgar and Primack 2011). Some species also have a chilling requirement and/or a critical photoperiod before buds can respond to spring warmth (Zohner 2016; Wang et al. 2020). Chilling accumulation is a primary predictor of the beginning of autumn senescence (Dufrene et al. 2005). Additionally, photoperiod can determine the onset date of chilling accumulation (Liu et al. 2024). Increasing autumn temperatures and summer drought may delay leaf coloration, but heat stress or heavy precipitation predict an advance in coloration, while heat stress or heavy precipitation predict an advance in coloration (Archetti et al. 2013; Xie et al. 2015, 2018).

Understanding what predicts leaf coloration is particularly important because it marks the period when carbon uptake is diminishing or has stopped (Wilson et al. 2000). Leaf coloration and drop may not be controlled by the same factors (Gill et al. 2015). Most studies of autumn predict leaf coloration, but understanding predictors of leaf drop phenology is also important as it determines the light received by understory species in autumn (Augspurger and Salk 2024). More phenological studies that encompass both spring and autumn phenology in warm and cold years, coupled with measures of net primary productivity, are necessary to determine their relative effects on carbon sequestration during climate change.

Earlier phenological events (or weather) may affect later events. These “legacy events” of phenology (or weather itself) from previous months or even previous years can be important predictors of later events (Keenan and Richardson 2015; Ettinger et al. 2018; Blonder et al. 2023; Prather et al. 2023). Warmer autumns may delay dormancy and slow advances in spring (Beil et al. 2021). The timing of bud dormancy in autumn can predict timing of spring leaf out (Malyshev et al. 2024).

Species have different thermal sensitivities to specific weather variables (Estrella and Menzel 2006; Vitasse et al. 2009). These differences have implications for species interactions, perhaps influencing relative abundances within the community. The specific requirements and phenological responses of most species are unknown and studies yield inconsistent results (Gill et al. 2015). Existing studies are largely of European tree species, including some species that occur in North America, but usually are not done in the context of one community (Laube et al. 2014; Zohner et al. 2016; Flynn and Wolkovich 2018).

Studies using satellite (Jeong et al. 2011), experiment (Fu et al. 2018), and observation (Fu et al. 2015) over recent decades (~1980–2010), despite regional and decadal differences (Jeong et al. 2011), have generally found a lengthening of the growing season that is due more to autumn delays than spring advances (Fu et al. 2015, 2018). However, non‐linearities in the amount of change due to temperature and precipitation variability have been found in long‐term, multi‐decadal studies (Fu et al. 2015; Jochner et al. 2016). Differences in the rates of winter and spring warming (Fu et al. 2015) and greater inter‐annual variation in weather may obscure long‐term patterns (Liu and Zhang 2020).

To deepen our understanding of the long‐term association of phenological responses with climate change, we collected long‐term, contemporary field phenological observations for a community of woody species in both spring and autumn in an intact mature forest in the Midwest, USA. Our focus is on community‐level trends, rather than species‐specific responses. The goals of the study were (1) to quantify leaf and flower phenological changes in spring, autumn, and the growing season duration of a community with 22 woody species of canopy trees, subcanopy trees, shrubs, vines, and tree saplings from 1993 to 2021, (2) to determine whether the length of the growing season is changing, and whether shifts in spring or autumn phenology are more responsible, and (3) to identify temperature and precipitation predictors of spring and autumn phenological events, and to determine long‐term trends in those predictors.

## 2 Materials and Methods

The study site was the north half of Trelease Woods, a 24.5‐ha temperate deciduous mature forest fragment dominated by *Acer saccharum* near Urbana, IL, USA. Topography is uniform with elevation varying by ±5 m. Observations were made of 17 phenological “events” (nine “stages”) in the annual life cycle of each of 15 dominant canopy tree species, two subcanopy species, two shrub species, two vine species, one vine genus, and saplings of three canopy species (Table 1). Tree species included in this study represented 96.3% of total basal area of trees greater than 22.6 cm DBH, based on data collected during a complete census of the north half of Trelease Woods in 2005 (J. Edgington, unpublished data). Sample size varied among species and declined over time due to mortality (mean = 13.2 individuals per species, range = 1–20 at beginning of study; mean = 7.4, range = 0–20 at end of study). Numbers of the three species of *Fraxinus* declined to zero near the end of the study and vines and shrubs decreased more than canopy trees and subcanopy trees. Species were included in analyses regardless of number of individuals or number of years with observations for individuals.

**TABLE 1:** Description of phenological events for each principal growth stage.

Principal growth stage	Event code	BBCH code^a^	Description of event
Bud swell	BS1	01	Bud 1/3 of final size
	BS2	02	Bud 2/3 of final size
	BS3	03	End of bud swelling; Bud fully swollen
Bud burst	BB1	07	Bud burst
	BB2	09	First green leaf tips visible
	BB3	10	Leaf/shoot emergence well beyond bud scales, but leaves not yet unfolding and entire petiole not visible
Leaf expansion	LE1	11	Individual leaf blade and petiole visible; leaf unfolded but not expanded; up to 33% of final size
	LE2	17	Leaf 67% of final size
	LE3	19	Leaf final size
Flowering	FLB	63	Begin Flower; 30% of flowers at full size and appear functional
	FLE	67	End Flower; 1 week after 70% of flowers appear non‐functional
Leaf senescence (Leaf coloration)	LC1	92	Leaves converted 33% of green to senescent color
	LC2	92^b^	Leaves converted 67% of green to senescent color
	LC3	93^b^	Leaf 100% senescent color
Leaf drop	LD1	93	33% of leaves fallen
	LD2	96	67% of leaves fallen
	LD3	97	100% leaves fallen

*Note:* Each “stage,” except Flowering, has a beginning date (1), intermediate date (2), and ending date (3), each date a so‐called phenological “event,” as well as its duration (see Appendix A: Table A4). Flowering has only beginning and end dates. See Appendix A: Tables A1, A2, A3 for summaries of specific criteria for each species. On a given census date, the event documented represents the tree crown as a whole, that is, the dominant condition of buds. Event codes are used throughout; standardized BBCH codes also are listed here as reference.

^a^ BBCH code follows Finn et al. (2007) (as modified from Meier et al. (2009)).

^b^ No clear event for LC2 and LC3 noted in Finn et al. (2007).

The predominant phenological status of each individual was determined using binoculars by the same observer weekly from mid‐February through June for spring events and September through December for autumn events from 1993 to 2021. *Aesculus glabra* saplings were observed in July–August for their early leaf coloration and leaf drop. Within the duration of each of five vegetative “stages”, three dates, reflecting a given stage being one‐third, two‐thirds, or three‐thirds completed on the individual, were noted, each date being a so‐called phenological “event” (Table 1; Appendix B: Text 1). Added criteria for events were based on a species' distinctive phenology (Appendix A: Tables A1, A2, A3).

For flowering phenology, only beginning (census during first week with 30% flowers open) and ending of flowering (first census after 70% flowers wilted) were recorded because flowering does not have clear developmental changes, and it is impossible to know, on a given census date, how long flowering will continue. Each event was not always observed because of rapid development within the 7‐day census interval; also, change from one event to the next sometimes took more than 1 week. Accordingly, an estimate of the date, based on simple linear interpolation, was made for each non‐observed event. The middle of three events (the “two‐thirds” event) were not used in any analyses, but were critical in determining estimated dates of non‐observed events.

Stage durations were based on event dates (e.g., Bud Swell Duration = [First DOY of Bud Burst] − [First DOY of Bud Swell]; Appendix A: Table A4). Flowering Duration was calculated as the difference between the DOY when 30% of the flowers were open, and 7 days after 70% of flowers were wilted. The two autumn stage durations each were calculated as (DOY of last event—DOY of first event). Total duration of Spring Phenology, Autumn Phenology, and Growing Season were also estimated (Appendix A: Table A4). Accuracy of a stage's duration is determined by census frequency. Greater error results with a greater interval between two censuses and during faster phenological development (Osteros et al. 2013). Given the length of the study, censusing at intervals shorter than weekly was not possible. However, caution in interpreting durations is necessary, especially for the fast‐developing stages in spring.

Our focus is largely on community‐level trends, rather than species‐specific responses. Instead of analyzing and presenting results for 22 species separately for 10 events and nine durations, we categorized species into different “seasonal groups”, with species within each group having similar mean timing of an event (Appendix A: Table A6). It was not desirable to build one model for all species combined because the date for a given event ranges widely among species that have been exposed to different weather conditions. Shorter gaps between the start of a weather metric (or the end of a cumulative weather variable, e.g., “CDD”) and an event date are preferred. This approach provides estimated responses of each species to the predominant weather predictors through random slopes (see below), although the predominant weather variables are determined at the seasonal‐group level and not for each species.

*Aesculus glabra* (Ohio buckeye or OB) saplings were a unique early seasonal group for almost all spring and autumn vegetative events; for Leaf Expansion, they were combined with OB canopy trees as one early group. OB canopy trees were a second unique early seasonal group for all autumn events. All other species were combined into a late seasonal group for all spring vegetative events through begin Leaf Expansion, but were separated into Intermediate and late seasonal groups for end Leaf Expansion and all autumn events (Appendix A: Table A5).

The mean number of days separating typical event dates within a seasonal group was 17.5 days (range = 12–26 days). Natural breaks of 4–6 days with no species active occurred for end Full Expansion and begin Leaf Coloration. Artificial breaks, used for end Leaf Coloration and both Leaf Drop events, divided the species roughly in half, with a range of about 15 days among species. For durations, each species stayed in the same seasonal group as the beginning event of a stage. Mean number of species in major seasonal groups was 13.5 (range = 3–24). For each combination of seasonal group and phenological measurement, 557–7111 records were in analyses with multiple species; number of individuals ranged from 38 to 327.

### 2.1 Weather Variables

Weather variables used in model selection to predict each event were based on literature for woody plants (e.g., Xie et al. 2015). Analyses included various combinations of mean daily, maximum and minimum temperatures, growing degree days (GDD for spring, also known as forcing accumulation) and chilling degree days (CDD for autumn and spring, also known as chilling accumulation), number of hot days, as well as total precipitation, number of rain and flood days (see Appendix A: Table A6 for details). Daily temperature and precipitation data from 1992 to 2021 were gathered from the Champaign, IL Weather Station (3S), 8 km SW of the study site. The station is part of the National Weather Service Cooperative Observer Program (US‐COOP, https://www.weather.gov/coop/) and data were obtained through the Midwestern Regional Climate Center (http://mrcc.purdue.edu/). The phenology of a given event may be dependent on earlier vegetative events (Ettinger et al. 2018; Augspurger and Zaya 2020). Therefore, dates of selected prior events were added as variables (“legacy events”), predicting subsequent events (see Appendix A: Tables A7a and A7b for details). Durations were not used as explanatory variables.

A specific combination of weather variables over varying time intervals was used for each event, depending on its timing (Appendix A: Tables A7a and A7b). Shorter gaps between the start of a weather metric (or the end of a cumulative weather variable, e.g., “GDD”) and an event date are preferred. Given the large quantity of analyses and the number of time intervals to cover for each event, we elected to use only 30‐ and 60‐day intervals. The first interval for accumulating a weather variable stopped at a “Stop Date,” the mid‐point in the range of interannual mean event date of species in a seasonal group, and worked backward either 30‐day (labeled as “m” for month) or 60‐day (labeled as “s” for season) intervals. Each subsequent weather variable began the day prior to the earliest day of the previous 30 days or 60 days. Values of the suffix for month and season are larger with a greater number of intervals back from the stop date (e.g., “Rain.m1” indicates rain days in Days 1–30 preceding the Stop Date, while “Rain.m2” indicates rain days in Days 31–60 preceding the Stop Date).

The number of temperature intervals was greatest (6 months and 3 seasons) for Begin Bud Swell and Begin Flowering to include possible chilling requirements in winter and forcing accumulation in spring and for autumn events in order to include weather from most of the growing season. Subsequent events used four 30‐day and two 60‐day intervals for mean temperature, growing degree days (GDD), chilling degree‐days (CDD), chilling degree‐days based on minimum temperature (CDD_i_), and frost days. Precipitation variables used a maximum of four 30‐day (two 60‐day) intervals. In addition to weather data from the previous 4–6 months, we used weather data from the previous growing season to predict event dates for four spring events: Begin Bud Swell, Begin Bud Break, Begin Leaf Expansion, and Begin Flowering. For weather predictors we used mean temperature and total rainfall from the previous year's summer (June–August) and autumn (September–November), as in Prather et al. (2023).

### 2.2 Statistical Analysis

Analyses for (a) temporal change in event start date, (b) temporal change of stage duration, and (c) weather and/or legacy variables that best predicted event date were tested using linear mixed‐effects models. Statistical methods were based on the methods of Xie et al. (2018) and Augspurger and Zaya (2020). All mixed‐effects models were constructed using R version 4.0.4 (R Core Team 2021) and the *lme4* package (Bates et al. 2015). All analyses were based on event dates observed on individual plants in each year, with stage durations calculated from these event dates. Standardized values of explanatory variables, scaled to have a mean of zero and standard deviation of one, were used in all analyses. However, raw (unscaled) values are used for result visualization, unless stated otherwise.

Nine events were analyzed for temporal change in event dates, five spring events and four autumn events (Appendix B: Text 1; Appendix C: Tables C1a and C1b). For each event, separate analyses were conducted for groups of species that had similar timing. Species were separated into two to four seasonal groups for each event, with a total of 27 combinations of event and seasonal group (11 spring; 16 autumn). A separate set of mixed‐effects models was created for each event‐season combination. The response variable was event date, measured as day of year. All models included random intercepts for individual plants. All models also included a random intercept for species (canopy trees and saplings of a single species were treated as separate groups). Random slopes for species were tested if an analysis had more than three species.

To test for temporal change in event dates, first, the fixed effects structure was chosen using model selection with the Akaike information criterion corrected for sample size (AIC_C_). Second, building from the optimal fixed effects structure, the optimal random effects structure was selected using the conditional AIC (cAIC; Greven and Kneib 2010), calculated with the *cAIC4* package (Saefken and Ruegamer, 2018; Saefken et al. 2018).

The temporal changes in the durations of nine stages were also tested (five in spring, four in autumn, and Growing Season spanning the two seasons; Appendix C: Table C2c). Seasonal groups were assigned based on the first event marking the stage. Each stage had 2–4 seasonal groups with a total of 24 combinations of stage and group. The same analytical approach was used for the durations of stages as described for event dates, except that the response variable was stage duration measured in days.

To test the influence of higher phylogenetic effects, we also constructed a set of temporal change models with genus, family, and order in the random effects structure for a subset of response variables. We found little to no improvement of model performance (∆AIC_C_ between −0.14 and 0.36) when using higher taxonomic classifications. Thus, we proceeded with models that included only species in the random effects structure and do not report results for the higher phylogenetic groupings.

For analyses of the relationship between event dates and weather variables, we tested the dates of seven events in 19 separate analyses based on seasonal groups of species (Appendix A: Tables A8a and A8b). These events and seasonal groups were the same as for the analyses of the temporal trend in event dates, except that End Leaf Coloration and End Leaf Drop were excluded because they are largely determined by the preceding event (“Begin” for each). In all analyses, the response variable was the event date, measured as day of year (DOY). Fixed effects consisted of weather variables and, in most cases, phenological variables that preceded the response (legacy effects). Weather variables described temperature and precipitation in multiple 30‐day intervals (“months”) or 60‐day intervals (“seasons”) preceding the typical date for an event within a seasonal group. The optimal fixed effects and random effects structures were chosen for each weather or legacy variable individually, using model selection with AIC_C_.

Models were created using weather and/or legacy variables in combinations of three or fewer variables, and compared with AIC_C_. Fixed effects included in the top model and at least four of the top five models were highlighted as important explanatory variables. For the weather variables chosen as important explanatory variables of a specific event date, we used linear regression to test for temporal change in the weather variable through time (1993–2021).

After we found the best model of weather and legacy variables predicting an event date, we repeated the tests of temporal change for the event date (linear mixed‐effects model) and weather variable (linear regression) for a shorter time period, 1993–2017; 2018–2021 had great variation in temperatures. Random slopes assigned to each species were used to determine relative thermal sensitivities.

In all three sets of analyses (temporal changes of event dates, temporal changes of stage duration, and weather or legacy variables that predict event dates), models were initially constructed using maximum likelihood for comparison with information criteria. Final model parameters were determined using restricted maximum likelihood. Confidence intervals for the species‐level coefficients were calculated using posterior simulations conducted with the *arm* package (Gelman and Hill 2007; Gelman and Su 2020). See Appendix B: Text 2 for further details on statistical modeling for all three groups of analyses.

Two approaches were used to evaluate models; see Appendix B: Text 3 for details.

## 3 Results

### 3.1 Spring Phenological Change

**FIGURE 1:**
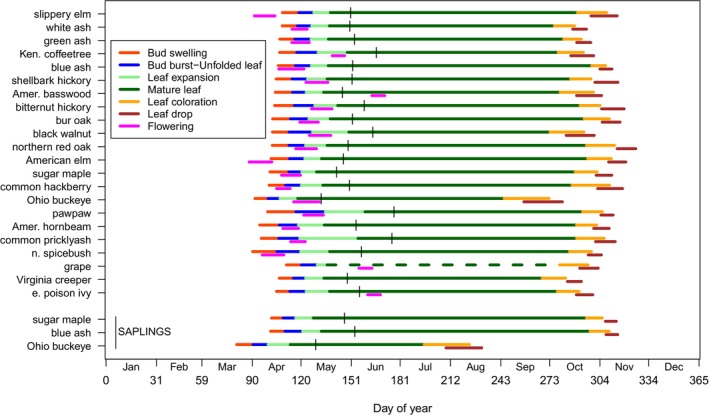
Annual phenology of each study species from 1993 to 2021 in Trelease Woods, Illinois, USA. See Appendix A: Table A5 for species nomenclature. Values plotted are mean dates per phenological event across all years; within a given year, the value for an event was calculated as the mean date across individuals of a given species. Species are ordered by mean date of beginning of bud swell, first by canopy tree species, then by treelets, shrubs, and vines, and finally by saplings of three canopy tree species. The vertical line indicates the date of full leaf maturity; the dark green line to the left of the vertical line indicates the period during which the fully expanded leaf is maturing. The dotted dark green line for grape indicates opportunistic growth of the vine for irregular periods of time. Not all species were used in analysis of a given event (see Appendix A: Table A5).

Early stages of spring phenology, based on means of 29 years, vary only about 2 weeks among canopy tree species, but become more variable as stages progress (Figure 1); *Aesculus glabra* is notably early. Non‐canopy tree species are more variable than canopy tree species, while saplings experience some period of beneficial high light as they predate conspecific canopy trees, markedly so in *Aesculus glabra* (Figure 1).

Flowering by most species, largely wind‐pollinated, is during Bud Burst and before Leaf Expansion, allowing the possibility of reducing leaf interference of moving pollen (Figure 1). Exceptions include the very early‐flowering, wind‐pollinated *Ulmus* species and insect‐pollinated, *Lindera benzoin* , and the later‐flowering *Juglans nigra* , *Gymnocladus dioicus* , and especially *Tilia americana* ; the latter two species are not wind‐pollinated.

**FIGURE 2:**
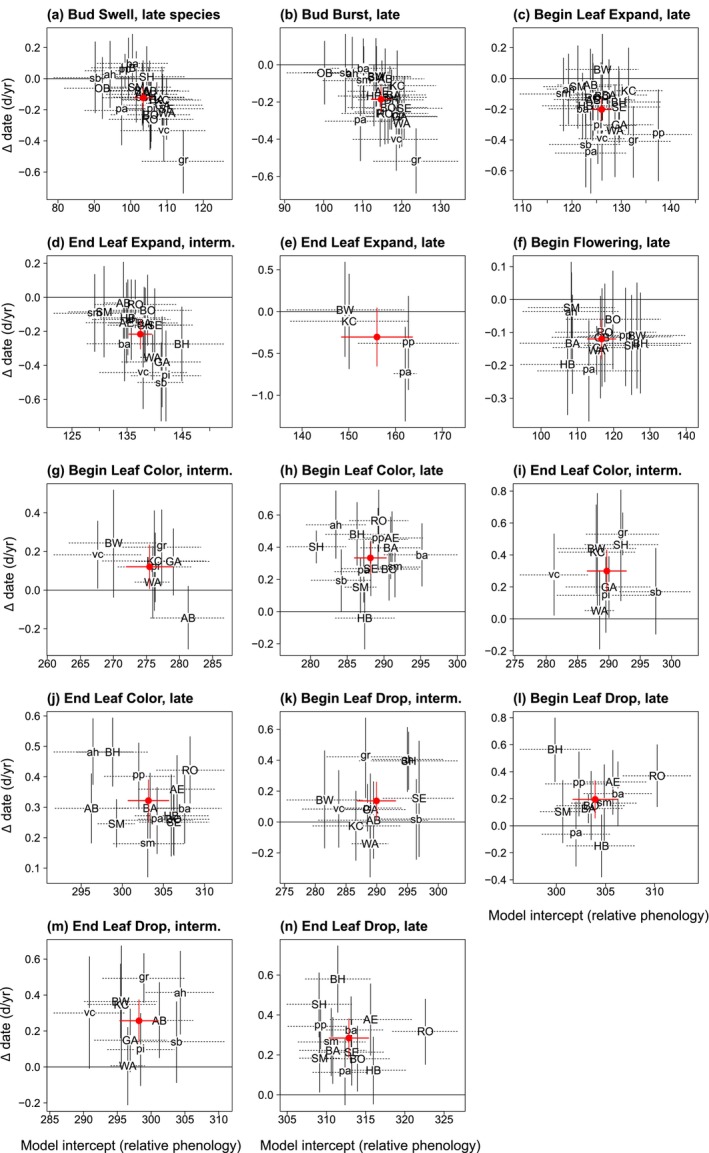
Change per year in 14 event dates versus relative phenology, for each species. Species are separated into seasonal groups for each event. Negative values on the *y*‐axis indicate earlier dates (advances), positive values later dates (delays). Values on the *x*‐axis indicate relative phenology (random species intercepts), where lesser values indicate species with earlier phenology. Solid vertical lines and dashed horizontal lines give the 95% bootstrap CI. Change in days represents the coefficient (random species slope, *b*) from linear mixed‐effects models, while the model intercept is the random species intercept from those models, as in the equation date = *b*(year) + intercept, where the year 1993 is set to year = 0. Two letters refer to abbreviation of the common name (see Appendix A: Table A5). The large red dot is the coefficient of the same variables from the seasonal group‐level analysis (±2 SE; red vertical and horizontal lines; Appendix C: Tables C2a and C2b). For red dots with no vertical SE, the top model did not include year as a fixed effect, that is, the event date did not change through time.

Over 29 years, beginning of spring events advanced for all 11 event–season combinations, except *Aesculus glabra* (Figure 2; Appendix C: Table C1a). Greater advance occurred with successive vegetative events, from 1.2 days/decade for Begin Bud Swell to 2.2 days/decade for End Leaf Expansion (Appendix C: Table C2a). For all species and events, 89.7% of spring events advanced, significantly more than expected at random (two‐sided exact binomial test, *p* < 0.00001; Appendix C: Table C3a). Advances for spring events result in a shorter time to full leaf expansion, and bring deep shade sooner to the understory.

For all spring events, except Begin (Late) and End (Late) Leaf Expansion, the degree to which a species shifted over time was correlated with that species' phenology (Figure 2; Appendix C: Table C1a). For example, Begin Bud Swell (Late) advanced more (y‐axis in Figure 2a, representing random species slopes) in species with later baseline Begin Bud Swell dates (x‐axis in Figure 2a, the random species intercepts). The pattern of species' differences in extent of advance results in less variation among species in spring event dates through time.

**FIGURE 3:**
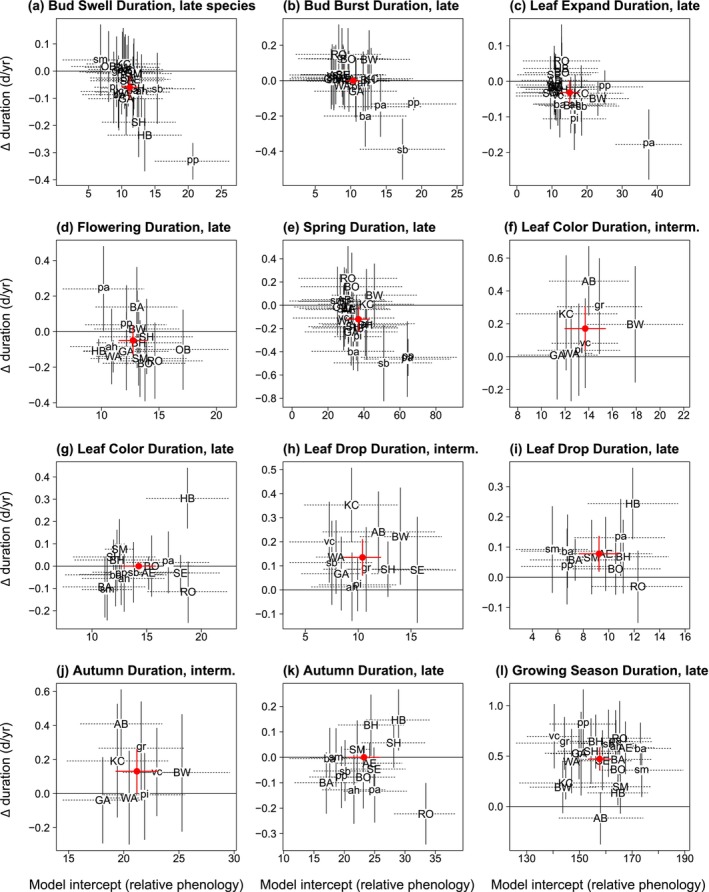
Change per year in 12 stage durations versus relative phenology, for each species. Negative values on the *y*‐axis indicate shortening durations, while positive values indicate lengthening durations. Values on the *x*‐axis indicate relative phenology (random species intercepts), where lesser values indicate species with shorter durations. See Figure 2 legend and Appendix C: Table C2c for additional details.

Durations of spring stages shortened or did not change, except Bud Swell Duration of *Aesculus glabra* saplings became longer (Figure 3; Appendix C: Table C3c). For all species and spring stages, 61.5% shortened, significantly more than expected at random (*p* < 0.007; Appendix C: Table C3c). Overall, Spring Duration shortened by a mean of 1.2 days/decade; 69.6% of species shortened (*p* < 0.029). For all four spring durations (Late), the degree to which a species shortened over time (y‐axis in Figure 3a) was correlated with its duration (x‐axis in Figure 3a; Appendix C: Table C4a). Species' differences in amount of shortening of duration may affect the degree of overlap among species, depending on the interaction with event date advances.

### 3.2 Autumn Phenological Change

Based on means of 29 years, a month separates canopy tree species in autumn events, except *Aesculus glabra* is notably early (Figure 1). Conspecific canopy trees predate saplings of *Acer saccharum* and *Fraxinus quadrangulata* , but saplings of *Aesculus glabra* greatly precede conspecific canopy trees. Non‐canopy species are somewhat earlier than canopy trees. The variation among species in the extent of overlap of timing of Leaf Coloration and Leaf Drop and large temporal differences among species are conspicuous (Figure 1), suggesting that the two processes are not tightly integrated and that all species either are not responding to the same environmental factor(s) or have different sensitivities to it.

Over 29 years, autumn events delayed for all event‐season combinations, except *Aesculus glabra* (Figure 2) (Appendix C: Table C1b). Delays ranged from 1.2 days/decade later for Begin Leaf Coloration (Intermediate) to 3.3 days/decade for Begin Leaf Coloration (Late) (Appendix C: Table C2b). For all species and events, 87.0% of autumn events delayed, significantly more than expected at random (*p* < 0.00001; Appendix C: Table C3b). These delays in most canopy tree species result in the deep shading of the understory persisting somewhat later.

Durations of autumn stages lengthened for six of eight stage‐season combinations (Figure 3; Appendix C: Table C2c) from 0.8 days/decade for Leaf Drop Duration (Late) to 3.8 days/decade for Leaf Drop Duration ( *Aesculus glabra* tree). Across all species, 84.0% of stages lengthened, more than expected at random (*p* < 0.00001; Appendix C: Table C3d). Total Autumn Duration did not lengthen for most groups. Among species, only 40% lengthened (*p* < 0.32).

For a given autumn event, the rate of change varied among species, but showed many more delays than advances (Figure 2; Appendix C: Table C3b). For all four autumn events, except End Leaf Coloration (Late) and all durations, the degree to which a species shifted over time was not correlated with that species' phenology (Appendix C: Tables C1b and C4b). Relative to spring, there is less similarity among species in autumn phenological responses to environmental factors and/or more variation in sensitivity to it.

### 3.3 Length of Growing Season

Length of Growing Season is determined by the duration between Begin Leaf Expansion and Begin Leaf Coloration. Overall, Begin Leaf Expansion advanced for 88% of species by a mean of a 2.0 days/decade from 1993 to 2021, while Begin Leaf Coloration delayed for 84% of species by a mean of 2.3 days/decade (Figure 2; Appendix C: Tables C3a and C3b). As a result, the growing season lengthened by 4.7 days/decade and for 88% of all species (*p* < 0.00007; Appendix C: Table C3d). Overall, the lengthening is due slightly more from autumn delays than spring advances.

**FIGURE 4:**
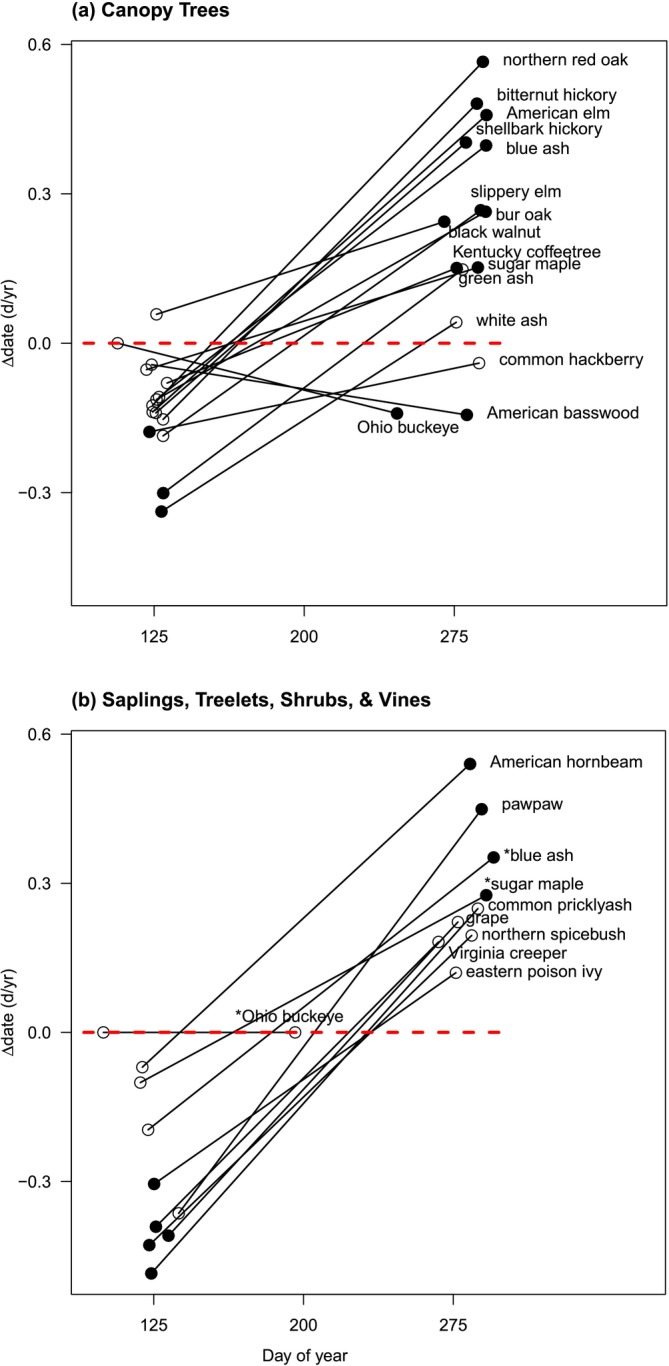
For each species of (a) canopy trees and (b) non‐canopy trees (* = sapling of canopy tree) over 1993–2021, a comparison of the number of days of change per year in spring (Begin Leaf Expansion; left dot) versus autumn (Begin Leaf Coloration; right dot) as a function of mean day of year of the event. Advances have negative values and delays have positive values. A black circle indicates the season that resulted in greater change in length of Growing Season; steeper slopes indicate a greater change in its length. Values were derived from each species' random slope coefficient for each event in the top mixed‐effects model (Appendix C: Tables C4a, C4b, C3c, C3d).

Non‐canopy woody species had a greater lengthening in growing season than canopy tree species (Figure 4). Non‐canopy species had a significantly greater advance in spring phenology (*t* = 2.5, df = 13, *p* = 0.028) rather than a greater delay at the end of the growing season (*p* = 0.585; Figure 4b; Appendix C: Tables C3a and C3b). This difference results in non‐canopy species having a longer time in high light in spring, but not in autumn, although with variation among species.

### 3.4 Weather Factors Predicting Phenology: 1993–2021

Among spring weather variables, “GDD” (forcing accumulation) had the strongest predictive power for 10 of 11 event–season combinations and was included in the top statistical model for all 11 events (Appendix C: Tables C5a and C6). “CDD,” an indicator of chilling accumulation, played a secondary role in models of the first spring event, Begin Bud Swell, and Begin Flowering (Late), suggesting that a chilling requirement may be a prerequisite in some cases before responding to forcing warm temperatures. A legacy event (Begin Bud Swell, Begin Bud Burst, or Begin Leaf Expansion) was the strongest predictor of five of 11 events, but “GDD” was the secondary variable in those same events. Precipitation variables, if included at all, were in secondary or tertiary roles in predictive models (Appendix C: Table C5a). When included, they were in only one of five top models, except “Rain.m3” was in all five top models for End Leaf Expansion of *Aesculus glabra* saplings and trees (Appendix C: Table C6). Temperature plays a much more key role than precipitation in predicting spring events.

**FIGURE 5:**
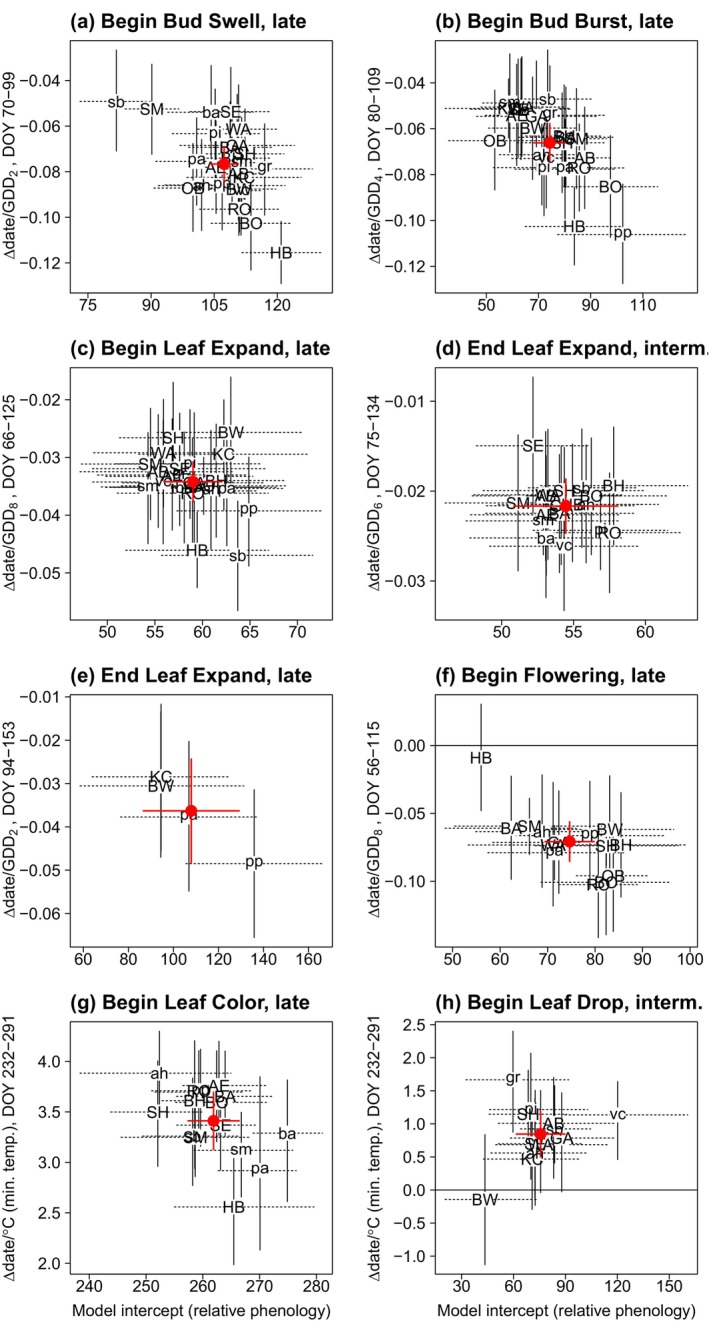
Change in date of seven events per weather variable unit (thermal sensitivity) versus relative phenology, for each species. Species are separated into seasonal groups for each event. Solid vertical lines and dashed horizontal lines give the 95% bootstrap CI. The relationship for a given event was included in this figure only if there was a weather variable that performed consistently as a predictor (Appendix C: Table C6) and random‐species slopes were included for that variable (Appendix C: Tables C4a, C4b, C10a, and C10b). Change in date per weather variable unit is the random‐species slope for the top model describing the relationship (Appendix C: Tables C7a and C7b), while relative phenology date is the random‐species intercept from the model. Smaller model intercept (x‐axis) values correspond with earlier phenological events. Letters refer to species' common names (see Appendix A: Table A5 for species and abbreviations). The large red dots represent the overall model coefficients for all species in the seasonal group‐level analyses (±2 SE; red vertical and horizontal lines; Appendix C: Table C6). All values in this figure represent the results of analyses based on untransformed (i.e., not standardized) values.

The spring phenology of all species advance in warm years or delay in cold years. For each of the strongest weather predictors for spring events, random slopes fit to each species all had a negative sign. Events advanced by a mean of 0.07 day/1‐unit GDD increase (range = 0.02–0.11 days; Appendix C: Tables C7a and C8). Species differ two to fivefold in their thermal sensitivity to the weather variables that best predicted community phenology (Figure 5).

Among weather variables in autumn, (CDD), a measure of chilling accumulation, had the strongest and most consistent predictive power for six of eight event–season combinations (Figure 5; Appendix C: Table C6). The legacy event of Begin Leaf Coloration was the top predictor for Begin Leaf Drop for all four event–season combinations, but a temperature variable was the secondary variable in those same events (Appendix C: Tables C5b and C6). Rain days played the primary role for *Aesculus glabra* saplings and drought was a secondary factor for *Aesculus glabra* trees in models for Begin Leaf Coloration; each was in the five top models (Appendix C: Table C5b). Precipitation variables were less influential for Intermediate/Late Groups. Begin Leaf Drop (Late) had no consistent weather predictor (Appendix C: Table C5b). Overall, the early autumn events that are potentially important for carbon gain are delaying as temperatures increase, while later events have less predictable patterns.

For most autumn events, species‐level slopes associated with the strongest weather predictor had primarily the same positive sign, indicating a delay in the event with an increase in temperature (Figure 5, Appendix C: Table C7b). However, the thermal sensitivity varied among species for each event–season combination, ranging from an advance of 0.1 day/1°C increase to a delay of 3.9 days/1°C increase. Additionally, there were large differences among seasonal groups; late species overall showed double the sensitivity in Leaf Coloration compared to Intermediate species, and the two groups had opposite reactions to warming for the timing of Leaf Drop (Appendix C: Table C8). Temperature variables for Begin Leaf Coloration for *Aesculus glabra* trees and Begin Drop (Late) had a negative sign (Appendix C: Table C8), indicating warmer conditions were associated with advances by a range of 0.38–2.03 days/1°C increase (Figure 5; Appendix C: Table A7b). As in spring, almost all species respond similarly to cold or warm temperatures in their autumn phenology, but with greater variation among species and greater thermal sensitivities.

**FIGURE 6:**
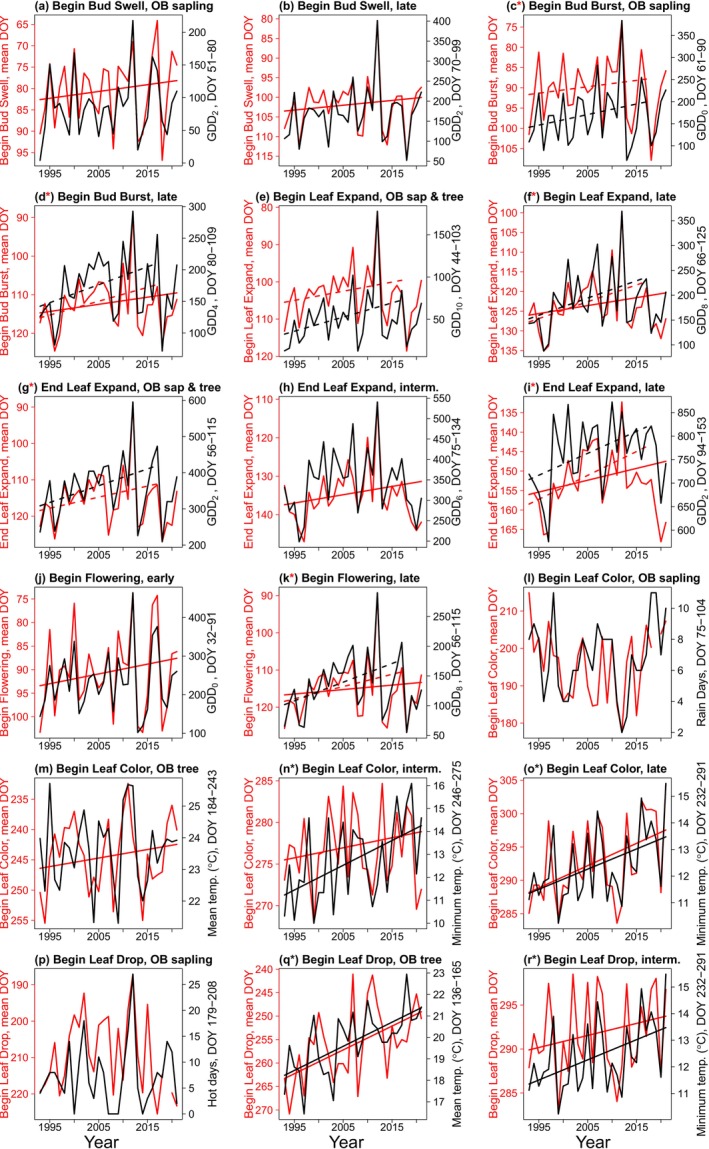
Inter‐annual variability in mean date of 18 phenological event‐by‐season combinations (red values) and the strongest weather model predictor for each (black values) from 1993 to 2021. Note that “OB” refers to Ohio buckeye, “Mean temp.” refers to mean daily mean temperature, and “Minimum temp.” refers to mean daily minimum temperature. When multiple weather variables were in the top five models (Appendix C: Tables C5a and C5b), the variable chosen had the highest absolute *t*‐value in the top model (Appendix C: Table C6). An inverted scale for the event date (left Y‐axis) was used for events that were negatively correlated with the weather predictor (all panels except L, N, O, and R). For weather variables, solid black regression lines represent the least‐squares regression through time when a significant trend was present (*p* < 0.05; Appendix C: Tables C9a and C9b), while no line indicates a non‐significant trend. For phenological events, solid red regression lines were created from the parameters in the change‐through‐time mixed‐effects model (Appendix C: Tables C1a and C1b); panels where red regression lines are absent represent cases in which year was not included in the top model (Appendix C: Tables C1a and C1b). Dashed lines show significant temporal trends from 1993 to 2017 for the weather variable (dashed black line) and event date (dashed red line) in cases with nonsignificant trends through 2021. A black asterisk (*) indicates that both phenology and weather variables have significant trends from 1993 to 2021, indicating support for the hypothesis that phenology has shifted with climate change. A red asterisk indicates that both phenology and weather variables had significant trends from 1993 to 2017, but not when including the last four years of data collection (driven by an extreme spring in 2018). End Leaf Drop (Late) is not presented because it had no weather variable in four of the top five models.

In spring, eight of 11 analyses showed a shift to earlier event dates (Figure 6). All spring events had a strong weather predictor consistently in the top models. However, no weather predictor changed significantly from 1993 to 2021 (Appendix C: Table C9a). Years from 2018 to 2021 had particularly strong variation in spring weather (e.g., Figure 6d). For analyses restricted to 1993–2017, six had a significant temporal change in spring event dates and weather predictors (Figure 6). Therefore, additional support for the association of many phenological events shifting with changing weather in spring was found for the years 1993–2017, although not when including 2018–2021.

Four of eight analyses had a strong weather predictor for autumn event date, and four more analyses had a strong secondary weather predictor when a legacy event was the strongest predictor (Appendix C: Table C6). Finally, four analyses had both a delay in phenology and a strong weather predictor that changed significantly through time (Figure 6; Appendix C: Table C9b). *Aesculus glabra* saplings with summer senescence were one exception as their predictor, Rain Days, was highly variable. The other exception was Begin Leaf Drop (Late) with no strong weather predictor. Therefore, additional support for an association of a change in autumn phenology with a change in weather was found for four of eight event–seasonal groups. Importantly, those four groups included 24 of 25 species–life form combinations. Thus, the strong shifts in phenology over this time interval are concordant with climate change.

### 3.5 Model Evaluations and Random Effects Variance

The majority of variance in the statistical models was captured by the random species intercepts. In all cases, there was greater (in one case equal) variance captured by species intercepts rather than the individual intercepts. Overall, the individual intercepts contributed little to the explanation of variance. Additionally, random species intercepts were much more influential than the random species slopes associated with weather predictors (for details, see Appendix B: Text 4; Appendix C: Tables C10a and C10b).

In model evaluations, model performance was similar for tests of temporal trends in event dates and temporal trends in stage durations. In evaluating the 19 models with weather or legacy variables predicting spring and autumn event dates, model robustness was improved with *R* ^2^ values that were approximately doubled (for expected values predicting observed values), and with lower root mean square errors (see Appendix B: Text 3 and Appendix D for detailed results of model evaluations with 10‐fold cross‐validation).

## 4 Discussion

### 4.1 Changes in Temporal Pattern

#### 4.1.1 Spring

Over the past three decades, this temperate deciduous forest experienced advances in the start of various vegetative spring events, from 1.2 to 3.3 days/decade. Flowering for most species advanced 1.2 days/decade. Among species, 95% of all spring events were earlier. To a lesser extent, durations of stages shortened. This extent of advancement is comparable to studies in the eastern USA (Yue et al. 2015) and European deciduous forests (Chmielewski and Rötzer 2001; Fu, Piao, et al. 2014), less than results from China (Ge et al. 2015), but greater than leaf‐out records of the USA National Phenology Network (Piao et al. 2019).

Plants living in cold winters develop a cold hardiness that prevents premature response to spring warmth. Generally, cold hardiness must be lessened before development can be forced by accumulating temperatures. Therefore, timing of bud break is affected by a chilling accumulation, the rate of loss of cold hardiness prior to bud break, and spring forcing temperatures (Kovaleski 2022). In this study, both late spring events and late species advanced more than early spring events and early species (Figure 2), reflecting the temporal acceleration in warmth and perhaps species' differences in rate of loss of cold hardiness or differences in thermal sensitivity.

In addition to changes of relative dates across species, there is the potential for changing of relative dates across events within a species, or “phenological decoupling” (Guo et al. 2023; Picornell et al. 2023). Phenological decoupling increases (or decreases) the time between stages. For example, earlier leafout can interfere with wind pollination (Buonaito and Wolkovich 2024). However, in this study the shift in timing of End Leaf Expansion and Begin Flowering for wind‐pollinated study species did not differ significantly (data not shown; *p* > 0.35).

Decreasing the difference in spring timing between late and early species decreases their difference in time to accumulate nutrients and carbon, affecting competitive ability (Roberts et al. 2015). Likewise, as in Klosterman et al. (2018), when spring warming started later, leaf development from budburst to leaf maturity in general became shorter. However, advances of earlier and shorter spring stages are of less physiological significance than later and faster spring stages because early leaves are not yet at leaf expansion, the period of rapid development of photosynthetic machinery and leaf size (Gu et al. 2003; Augspurger et al. 2005). Faster leaf expansion shrinks the time to form chloroplasts, accumulate chlorophyll, and manufacture all apparatus for photosynthesis during leaf development (Jiang et al. 2006). If this process is completed rapidly (and therefore sooner) in spring, a longer period at peak photosynthesis rates arises, potentially increasing the individual plant's carbon storage, depending on respiratory costs (Gu et al. 2003).

Faster leaf expansion also lessens the time to leaf maturity, resulting in less time for herbivores to feed during the leaf's most vulnerable expanding leaf stage (Wiggins et al. 2016). Also, a greater advance by a late species may bring an advantage in interspecific competition. Importantly, at the ecosystem level, the timing of spring stages has a large effect on early‐season productivity and carbon sequestration of deciduous forests. In an advanced spring, carbon uptake via photosynthesis can exceed its loss via respiration (Keenan et al. 2014), but see below.

Advances of spring stages and rapid leaf development are not always positive. Plants can respond to a “false spring,” that is, when particularly early warmth initiates development that is later damaged by a cold period (Vitasse et al. 2018). Studies have found that expanding buds are least cold hardy, while expanding leaves are slightly more resistant to freezing (Lenz et al. 2013, Chamberlain et al. 2019; North and Kovaleski 2024). However, warmer winters with shorter chilling can delay leaf‐out, extending the period of frost risk (Chamberlain and Wolkovich 2021). At our study site, record‐breaking warm temperatures in March 2007 brought rapid leaf development, followed in early April by a record‐breaking freeze that destroyed many expanding leaves. Buds were not damaged more than expanding leaves because most buds had opened and thus were not exposed to the freeze. Re‐foliation occurred, but brought delayed full leaf expansion (Augspurger 2009). Frost events of differing severity were common throughout the study. The combination of spring warming and temperature extremes may be becoming more common (Augspurger 2013), which may threaten individuals of particular species and life forms (e.g., *A. glabra* saplings, vines in general) with greater damage or mortality (Augspurger 2011).

#### 4.1.2 Autumn

All seasonal groups, except canopy trees and saplings of *Aesculus glabra* , delayed the start of autumn events from 1.2 to 3.3 days/decade and, to less of an extent, lengthened durations, especially leaf drop in some species. Among species, 87% of autumn events were delayed. This study's delay of leaf coloration is comparable to Chinese (Ge et al. 2015) and European trees (Menzel et al. 2020). In a world‐wide analysis, leaf coloration in deciduous forests delayed 3.3 days/decade (Gill et al. 2015).

Carbon gain depends in part on how long plants can stay at their peak level of photosynthesis as autumn's leaf coloration approaches (Gu et al. 2003). Generally, the amount of photosynthesis per unit time declines from mid‐ to late‐summer onward because of the start of breakdown of its machinery (Krupinska and Humbeck 2004; Augspurger et al. 2005). Loss of chlorophyll is correlated with temperature and day length, and is connected to various hormones triggering senescence (Zhang, Dai, and Ge 2020). An increase in growing season productivity may promote earlier leaf coloration (Zani et al. 2020). This study's results of a delay in leaf coloration may reflect a delay (or slower rate) of the breakdown of photosynthetic machinery. Any delay thus may provide a longer, continuous period of photosynthesis, although a lower sun angle in autumn than spring brings less solar radiation for photosynthesis (Zhang, Commane, et al. 2020). A longer duration of leaf coloration also may indicate a longer period for nutrient remobilization from leaves (Calinger and Curtis 2023). Sequestration of nitrogen before leaf drop and nitrogen storage is critical for manufacturing photosynthetic machinery in new spring leaves, and for spring growth (Jordan et al. 2012; Estiarte and Peñuelas 2015).

Unlike spring events, the amount of delay for autumn events was generally independent of when species started leaf coloration. Species delaying leaf coloration may experience greater competitive ability by accumulating more net carbon and nutrients, although that advantage may be tempered by decreased available radiation in autumn (Zhang, Commane, et al. 2020).

### 4.2 Length of Growing Season

A longer growing season is the major consequence of these spring advances and autumn delays. From 1993 to 2021, the growing season in this study's forest community lengthened by 4.7 days/decade for all species, excluding the early *Aesculus glabra*. Few long‐term studies of both spring and autumn changes for a forest community are available to compare recent growing seasons; most focus on only spring phenology.

The growing season lengthening is due more to a delay of Begin Leaf Coloration (2.3 days/decade) than advance of Begin Leaf Expansion (1.9 days/decade), very similar to estimates for Continental Europe (Garonna et al. 2014). Both experimental (Fu et al. 2018) and satellite (Garonna et al. 2014) studies in Europe have found a greater delay in autumn than an advance in spring leaf out in recent decades. As in this study, the lengthening of the growing season in mid and high latitudes in North America from 1982 to 2006 was due to delayed autumn and not advanced spring (Zhu et al. 2012).

A complete picture of the effect of long‐term changes in phenology requires more than quantification of the length of the growing season. The relative contributions of timing and speed of leaf photosynthesis and respiration, driven by changes in phenology in both spring and autumn, need to be modeled to better understand the significance of both advances and delays of leaf phenology in temperate deciduous forests. The capacity of the forest vegetation to assimilate CO_2_ differs between a comparable advance in spring and delay in autumn. An advanced spring can result in positive net carbon gain (Keenan et al. 2014). However, during a delay from warmer autumns and a longer growing season, carbon uptake via photosynthesis can be much less than carbon loss via respiration (Dunn et al. 2007; Piao et al. 2008).

Overall, the lengthening of the growing season is potentially important at multiple spatial scales. Globally, it may lessen carbon atmospheric input from biological processes, including respiration (Polgar and Primack 2011). Regionally, it affects immigration and species' ranges (Morin et al. 2007), thereby affecting community assembly and changing local species composition with different temporal niches (Morin and Thuiller 2009; Wolkovich and Donahue 2021; Ponti and Sannolo 2023). Locally, it changes the period of ecosystem services by canopy trees, including productivity (Richardson et al. 2010), water run‐off (Chen et al. 2022), and nutrient absorption and later remobilization (Estiarte and Peñuelas 2015). Also, it potentially impacts species' demography (Iler et al. 2021), population size, intra‐specific and inter‐specific competition (Fridley 2012), and interactions with higher trophic levels (Rudolph 2019; Robertson et al. 2024). Finally, it expands the potential influence by the canopy on the light environment of understory species (Augspurger and Salk 2024; Xiong et al. 2024).

### 4.3 Weather Predictors

Identifying a weather predictor strengthens the claim of an association between phenology adjustments and climate change. Also, it gives insight into a possible environmental control of an event, and offers where to explore its exact mechanism (Kðrner and Basler 2010).

#### 4.3.1 Spring Phenology

The strongest predictors of spring events were predominately temperature variables, expressed as GDD (cumulative forcing) of different thresholds, or earlier legacy events within the same season. Accumulating warmth of the preceding month or 60 days predicted earlier event dates, but, in contrast to Fu, Campioli, et al. (2014), the association between prior year's weather predictors and spring events was relatively weak. Overall, these results are similar to studies in the Northeast USA (Richardson et al. 2006), Europe (Menzel et al. 2006), and Asia (Geng et al. 2020). When a legacy event was the strongest predictor, a temperature variable was the second strongest predictor. Precipitation was not a strong predictor for any spring events. Weather variables from the prior year were not among the most predictive variables of spring event dates (but see Blonder et al. 2023; Prather et al. 2023).

Our analyses were based on groups of species. It is possible that individual species' analyses would provide greater resolution of weather variables' influence on phenology, as well as interactions between weather variables. However, studies differ in their findings, even for individual species.

Multiple environmental variables likely interact to determine a species' phenology. Photoperiod and winter chilling, in addition to temperature forcing, can be required for initiating spring phenology (Basler and Kðrner 2012; Flynn and Wolkovich 2018). Fixation on a direct temperature control of phenology may obscure how mechanisms may interact to influence phenological trends. For example, some spring events had CDD (chilling accumulaton) as a secondary predictor, perhaps related to an additional chilling requirement over winter prior to responding to spring warmth (Wang et al. 2020; Zhang et al. 2022). Additionally, patterns of warmth in winter and spring may affect leaf unfolding rather than earlier events (Zhang et al. 2022). Greater warmth (less chilling) in autumn may delay induction of dormancy, thus resulting in less advance in spring events (Beil et al. 2021). While spring events advanced in this study, the non‐experimental methods cannot evaluate if the extent of advance was affected by delayed dormancy. Warming in both winter and spring may result in both a delay in reaching the chilling requirement and an advance from forcing warm temperatures (Ettinger et al. 2020). With these opposing forces, it is difficult to determine the exact factor(s) influencing spring phenology.

Photoperiod control has been studied for some temperate trees. An extensive experimental study found that day length limited responses to climate change in a minority of Northern Hemisphere species (Zohner et al. 2016, but see Flynn and Wolkovich 2018). Shrub species tended to show smaller budburst responses to photoperiod than many tree species, but this was not seen for leafout (Flynn and Wolkovich 2018). Focusing on 12 study species with European (Basler and Kðrner 2012; Laube et al. 2014; Zohner et al. 2016) and North American (Flynn and Wolkovich 2018) experimental studies that included photoperiod control, the four study species with high photoperiod sensitivities, *Quercus macrocarpa* , *Quercus rubra* , *Acer saccharum* , and *Carya cordiformis* , each showed a weak temporal trend, but not near constancy, of dates of spring events through 29 years. Temperature may have a greater influence than photoperiod on timing of spring phenology for these species, although interactive effects among environmental cues can be large (Flynn and Wolkovich 2018).

#### 4.3.2 Autumn Phenology

Similar to Archetti et al. (2013), the major predictor of the delay in Begin Leaf Coloration was minimum temperature of September/October. Precipitation variables were of secondary or tertiary importance for almost all species. Identifying possible drivers of phenology is dependent on the range experienced within a weather variable. During this study period, most species did not experience heavy rainfall or heat stress, which Archetti et al. (2013) and Xie et al. (2018) found to be important variables predicting autumn phenology in northeastern forests of the USA. Only the very early coloration of *Aesculus glabra* saplings and canopy trees included drought and hot summers as predictors. Contrasts in precipitation during the study were greater than temperature during summer and early fall and may explain this species' dependence on these changes as triggers to initiate leaf senescence.

Begin Leaf Drop was most strongly predicted by the legacy event of Begin Leaf Coloration, followed by minimum temperature, as in Xie et al. (2018). As noted by Ettinger et al. (2018), earlier “legacy events” can be stronger predictors than weather variables of many subsequent events, but only events within the same season. While this study indicates an increasing delay in autumn senescence over recent decades, predicted by warmer temperatures 1 month earlier, further exploration of earlier season(s) factors is warranted. Unlike Keenan and Richardson (2015) in the Northeastern USA and experimental studies of Fu, Campioli, et al. (2014), warming inducing an earlier spring at this local scale was not among the strongest predictors of earlier Leaf Coloration. Studies of productivity prior to autumn are needed (Zani et al. 2020), as well as comparisons of the rapidity of warm versus cold years on spring development and speed of development to the end of senescence (Zohner et al. 2023).

### 4.4 Association With Climate Change: Integration of Weather and Phenology

A strong weather predictor indicates it is highly correlated with the timing of a specific phenological event, but does not provide evidence of its direct causation of the event. By our taking the additional step of determining that both the phenological event date and weather predictor show parallel (or inverse) linear change through time provides further evidence of a direct association between phenology and climate change (Menzel et al. 2006). Analyses indicating temporal change in both weather and phenology for many woody species in a North American deciduous forest have not been done.

In this study of woody species, the temporal pattern of autumn, but not spring, events did parallel the temporal patterns of specific weather predictors over the period 1993–2021. However, by restricting the analyses for spring to 1993–2017, spring events also showed weather and phenology concordance. Interannual variation of temperatures is increasing (Liu and Zhang 2020). During our study, February and March had high interannual variation that included some extreme weather years, particularly from 2018 to 2021, thereby obscuring some long‐term patterns (Liu and Zhang 2020).

For three of four major autumn event‐season combinations, concordance between weather and phenology is strong because their predictor, September minimum temperatures, increased significantly over all the study's years, while autumn events were delayed. In the Midwest, USA, minimum temperatures have increased more than maximum temperatures from 1901–1960 to 1986–2016 (Wuebbles et al. 2017). The exception for autumn concordance was Begin Leaf Drop for the few Late species with no strong weather predictor. These late species show erratic patterns of leaf drop, with leaves withering, but not always dropping soon thereafter. In addition, some species have a very slow senescence, sometimes even dropping green leaves after a very late frost. It is unknown whether negative consequences arise from these atypical responses. Perhaps they affect the amount of nutrients mobilized from leaves, thus affecting nutrients needed for leaf development in spring (Jordan et al. 2012).

### 4.5 Thermal Sensitivities and Species‐Specific Traits

Within a given seasonal group, most or all species showed the same pattern of advance or delay of events. Durations showed more inter‐specific variation than events. However, the study species shift a given event at different rates and a given species shifts different events at different rates. *Aesculus glabra* is a clear outlying species in both its early spring and autumn phenology, especially its saplings. This species has different timing and sensitivity, as well as dependence on precipitation rather than temperature. Their saplings show growth and survival benefits from the high light of early spring (Augspurger 2008), but are vulnerable to late spring frosts (Augspurger 2011).

In general, species differ in the environmental factor(s) related to their phenology. Some may be sensitive to drought, photoperiod and/or winter chilling, have a low sensitivity to temperature change, or lack adaptive plasticity to respond. Thermal sensitivities in spring vary among the study species two‐ to fivefold (Figure 5), depending on event, similar to European trees (Vitasse et al. 2009). For early events, earlier species don't respond to warming as much as later species. Perhaps early species are vulnerable to frost and have a greater chilling requirement and/or lower thermal sensitivity to initiate spring growth, thus responding slower to warming.

Differences in sensitivities among species regulate the extent of species' overlap in spring and/or autumn. In turn, species‐specific differences in phenological change may result in shifting biotic interactions (Vitasse et al. 2009), including interspecific competition (Roberts et al. 2015). Some species may become winners and others losers as climate change continues, leading to changing communities (Diez et al. 2012).

Functional traits (Panchen et al. 2014), as well as phylogeny (Willis et al. 2008), may explain some species' differences in phenology. The study species differ in phylogeny, leaf size and complexity, determinacy of shoot development, and xylem anatomy, as well as light environment of different life stages, and growth forms. Although sample size limits statistical analysis, trends related to some traits were evident and confirm earlier studies.

First, phylogenetic relationships were not important in explaining phenological differences among the limited diversity of species. Second, *Gymnocladus dioicus* and *Juglans nigra*, with the latest End Leaf Expansion (and longest Duration of Leaf Expansion), have doubly compound and simple compound leaves, respectively, and the greatest median leaf lengths among our study species. Third, exceptionally late End Leaf Expansion occurred for *Asimina triloba* and *Zanthoxylum americanum*, both understory species with indeterminate shoots that continue to expand opportunistically in high light. Fourth, as first noted by Lechowicz (1984), xylem anatomy is associated with phenology. Begin Bud Swell and Begin Bud Burst were earlier by 9–10 days for the four species with diffuse‐porous xylem (*Aesculus glabra, Carpinus caroliniana, Acer saccharum*, and *Tilia americana* ) than the other species, all of which have ring‐ or semi‐ring‐porous anatomy. The large diameter vessels of ring‐porous species are subject to cavitation; their repair to begin water transport in spring results in later bud swell/burst (Lechowicz 1984). Finally, saplings predated conspecific canopy trees in spring events for all three species, but only for *Aesculus glabra* in autumn events. These results confirm a broader, earlier study in the same forest (Augspurger and Bartlett 2003). Temperature differences at the two levels of the forest (Augspurger 2004) or ontogenetic changes (Vitasse 2013) may explain the differences in timing between life stages in the understory versus the canopy.

Significantly, this is the first study to show that growth forms were mismatched in which season lengthened the growing season more. Canopy trees had a greater delay in autumn than advance in spring. In contrast, non‐canopy woody species had greater advance in spring than canopy trees, but did not differ from trees for the end of the growing season (Figure 4). Growing season lengthened more for non‐canopy woody species than canopy tree species. The difference in timing potentially provides more light for understory woody species. In contrast, Ge et al. (2015) found no differences in phenology between trees and shrubs from 1960 to 2011.

### 4.6 Other Applications

An additional study (Augspurger and Salk 2024) compared this study's data on woody species to that from a comparable, contemporary phenological study of the Trelease Woods community of 33 herbaceous species (Augspurger and Zaya 2020). Light availability in the understory from 1995 to 2021, resulting from phenological change of canopy tree species, was directly quantified. Most herb species and saplings adjusted their phenology comparable to canopy tree species, with little change in light to the understory (Augspurger and Salk 2024). Only a minority of these understory species have been receiving less light with the trend of increasing warming in the past 2.5 decades. Gross carbon gain by four of the study's herb species showed patterns similar to light patterns over the decades. Ultimately, changes in light available to more species of herbs (Heberling et al. 2019) and woody understory species need to related to gains in net carbon assimilation.

Data from this study have been used as ground‐truthing of remotely sensed imagery of spring canopy phenology at the study site. First, the framework developed by Zhao et al. (2023) effectively bridged the satellite‐ and field‐based phenological measures for Budswell, Bud Burst, and Leaf Expansion events at the individual tree‐crown scale, particularly for large individuals. Bud Burst/Leaf‐out and early Leaf Expansion events were retrieved with highest accuracy. Diao et al. (2024), using a satellite‐field phenological bridging framework that excluded effects from understory, soil and snow, found that key spring stages, summarized at the community level, were detected with high accuracy.

## 5 Conclusions

This study confirms worldwide studies indicating that climate change is associated with a longer growing season of temperate deciduous forests. It qualifies that phenological stages, growth forms, and species differ in the extent of their responses. It contrasts with most phenological studies of temperate deciduous forest by being in North America, long‐term, community‐based, including multiple growth forms in a natural, mature forest, and including both spring and autumn events. Additionally, no study of a North American mature forest community shows that long‐term trends in phenology and weather predictors are parallel in spring (1993–2017) and autumn (1993–2021), thus strengthening the evidence of a connection between climate change and phenology. Unexpected differences in phenology between strata of woody species were found as understory species advanced in spring more than canopy species.

This study adds to limited prior studies showing an acceleration of leaf development during spring stages. It confirms that the lengthening of the growing season is due more to autumn delays than spring advances. The importance of legacy events of the current year in predicting some future events is shown, as well as unique functional traits associated with a species' phenology. The study provides long‐term phenological data from a new geographic region in North America.

The study contributes data on the canopy duration in a mature deciduous forest, a measurement so vital to models of carbon sequestration, forest productivity, and other ecosystem processes (Weiskopf et al. 2020). More studies are needed now to quantify the relative contribution of changes in spring versus autumn phenology to carbon sequestration. Additional questions can be addressed with the current data set, for example, extent of variation among individual plants in their phenological response to climate change (Marchand et al. 2020), inter‐annual comparisons of date versus rate of spring green‐up (Klosterman et al. 2018), influence on species synchrony of green‐up with rapid temperature increase (Wang et al. 2016), relationships among stages and extent and mechanism by which legacy events predict future events long‐term (Ettinger et al. 2018), and relative changes among species with different latitudinal distributions (Morin et al. 2007). Finally, the complexities of phenological responses among species point out the need to better understand genetic and physiological mechanisms underpinning each species' phenology, thermal sensitivity, and integration of events within and between seasons (Wilczek et al. 2010).

## Author Contributions

**Carol K. Augspurger:** conceptualization (lead), data curation (lead), investigation (lead), methodology (lead), project administration (lead), writing – original draft (equal), writing – review and editing (equal). **David N. Zaya:** data curation (supporting), formal analysis (lead), validation (lead), visualization (lead), writing – original draft (equal), writing – review and editing (equal).

## Conflicts of Interest

The authors declare no conflicts of interest.

## Figures and Tables

**FIGURE D1:**
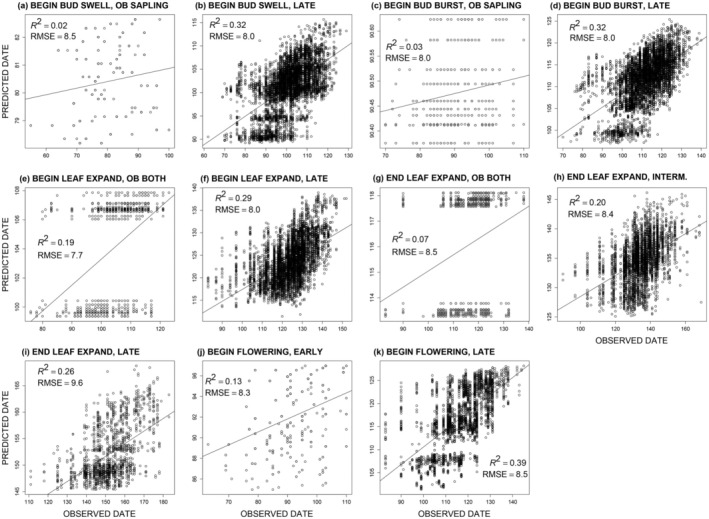
Validation of top temporal models for five spring phenological events of woody species at Trelease Woods (1993–2021) (A–K). Species are separated into two to three seasonal groups for each phenological event, yielding 11 event–season combinations. *R* ^*2*^ values indicate the coefficients of determination for observed values and the predicted values from the model. We used 10‐fold cross‐validation to evaluate model fit, and report the root‐mean‐square error (RMSE). Trend lines represent the least‐squares regression of the predicted and observed values.

**FIGURE D2:**
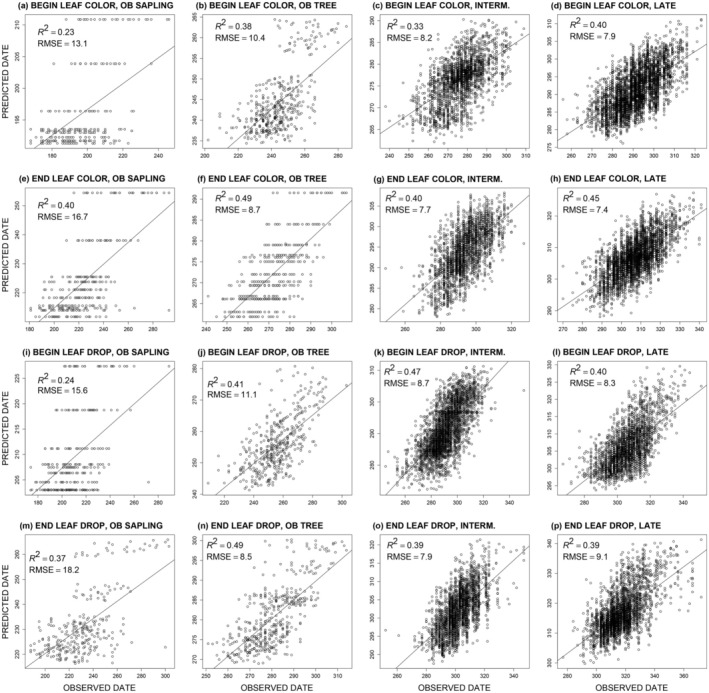
Validation of top temporal models for four autumn phenological events of woody species at Trelease Woods (1993–2021) (A–O). Species are separated into 2–4 seasonal groups for each phenological event, yielding 16 event‐season combinations. *R* ^*2*^ values indicate the coefficients of determination for observed values and the predicted values from the model. We used 10‐fold cross‐validation to evaluate model fit, and report the root‐mean‐square error (RMSE). Trend lines represent the least‐squares regression of the predicted and observed values.

**FIGURE D3:**
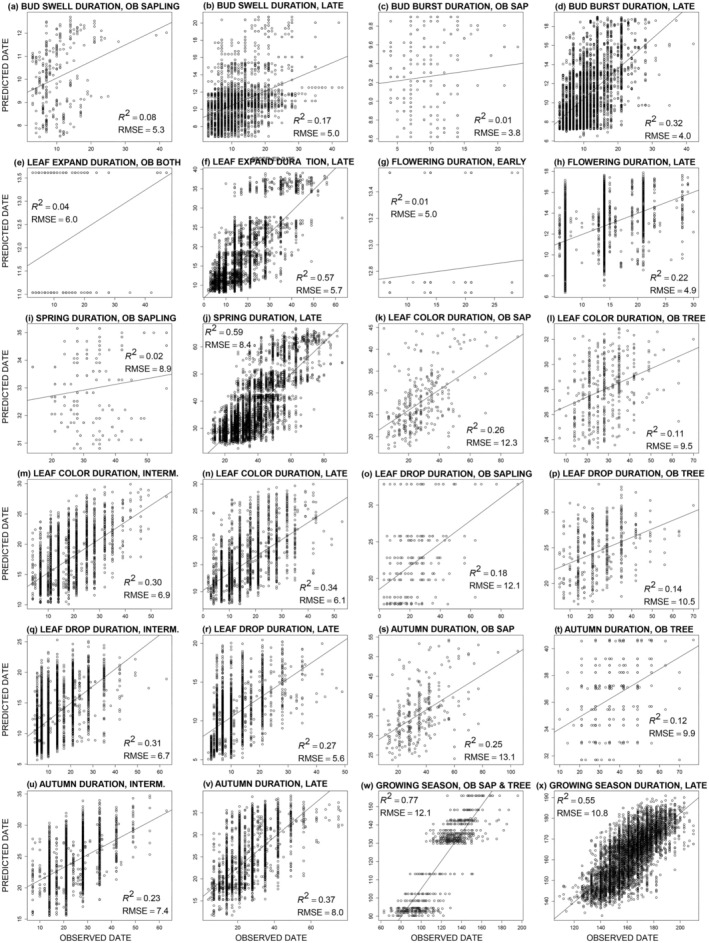
Validation of top temporal models for nine durations of phenological stages of woody species at Trelease Woods (1993–2021) (A–X). Species are separated into 2–4 seasonal groups for each stage, yielding 24 stage–season combinations. *R* ^*2*^ values indicate the coefficients of determination for observed values and the predicted values from the model. We used 10‐fold cross‐validation to evaluate model fit, and report the root‐mean‐square error (RMSE). Trend lines represent the least‐squares regression of the predicted and observed values.

**FIGURE D4:**
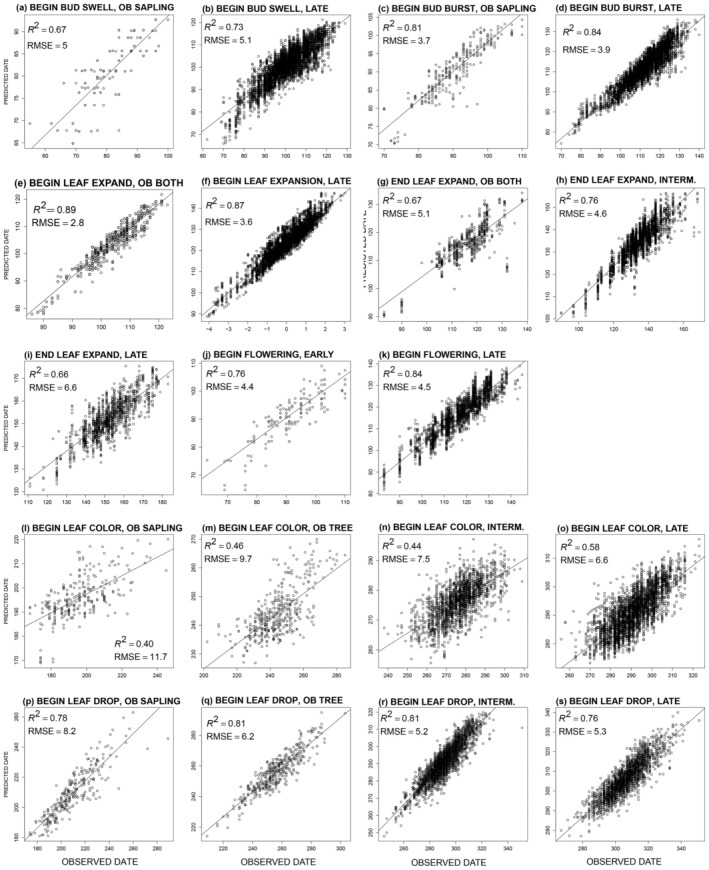
Validation of top weather models predicting seven phenological events of woody species at Trelease Woods (1993–2021) (A–S). Species are separated into 2–4 seasonal groups for each phenological event, resulting in 19 event–season combinations. *R* ^*2*^ values indicate the coefficients of determination for observed values and the predicted values from the model. We used 10‐fold cross‐validation to evaluate model fit, and report the root‐mean‐square error (RMSE). Trend lines represent the least‐squares regression of the predicted and observed values.

## Data Availability

Data are provided in: Augspurger, C. K. and D. N. Zaya. 2024. Woody Phenology and Weather Data related to Trelease Woods, Urbana, IL, USA, 1993–2023, Dryad, Dataset, https://doi.org/10.5061/dryad.3j9kd51p1 (Augspurger and Zaya 2024).

## Appendix A

**TABLE A1:** Description of criteria used to define first event (BS1) of Bud Swell stage for each study species.

Species	Criteria
*Tilia americana* Amer. basswod	Green showing between/above bud scales
*Ulmus americana* Amer. elm	Green showing between/above bud scales; occurs when change from flowering to early development of fruits
*Carya cordiformis* bitternut hickory	No bud scales so code BS1 when see swelling of terminal structure on stem with binoculars
*Juglans nigra* black walnut	Bud swell of gray buds noticeable (measure bud size for BS 1,2,3)
*Fraxinus quadrangulata* blue ash	Developing flowers obscure leaf bud area; flower/leaf buds open synchronously; coded BS1 when first see swollen green bud through flower structures
*Quercus macrocarpa* bur oak	Bud swell noted when bud becomes distinct and changes from brown to white/cream (measure size of bud for BS 1,2,3)
*Celtis occidentalis* common hackberry	White furry bumps denote BS1 (not tiny bumps invisible without binoculars and not green bumps)
*Fraxinus pennsylanica* green ash	Developing flowers obscure leaf bud area; flower/leaf buds open synchronously; coded BS1 when first see swollen green bud through flower structures
*Gymnocladus dioicuus* Kentucky coffeetree	Brown buds swell out of branch. Harder to see until full size (BS3)
*Quercus rubra* northern red oak	Bud swell noted when bud becomes distinct and changes from brown to white/cream (measure size of bud for BS 1,2,3)
*Aesculus glabra* Ohio buckeye	Buds swell noticeably and scales turn red/cream (measure size of bud for BS1,2,3)
*Carya laciniosa* shellbark hickory	White scales/bud swollen so change in size is visible from ground without binoculars for BS1
*Ulmus rubra* slippery elm	Green showing between/above scales for BS1
*Acer saccharum* sugar maple	Greenish/silvery brown showing between scales but no leaves out of bud scales for BS1
*Fraxinus americana* white ash	Developing flowers obscure leaf bud area; flower/leaf buds open synchronously; coded BS when first see swollen green bud through flower structures
*Carpinus caroliniana* American hornbeam	Size of bud does not change much; green showing between bud scales for BS1
*Asimina triloba* pawpaw	First bud scale separating and bending outward = BS1. 1 or 2 scales can do this early and then nothing happens for some weeks when swelling also occurs (BS2 and BS3)
*Zanthoylum americanum* common pricklyash	Yellowish‐cream showing between bud scales = BS1; not swollen brown flower bud
*Lindera benzoin* northern spicebush	Green showing between or above bud scales = BS1
*Toxicodendron radicans* eastern poison ivy	No obvious color change from gray bud; measure as BS 1,2,3
*Vitis* spp. grape	Green buds swell and emerge from branch; hard to see in early stages so much estimation of BS1
*Parthenocissus quinquefolia* Virginia creeper	Obvious color change to red as bud swells and new parts of bud scales appear

*Note:* See Table A4 for names of events abbreviated below.

**TABLE A2:** Species‐specific issues and solutions in spring vegetative and flowering events and phenological stages.

Vegetative events	
Species	Issues and solutions
*Ulmus americana* American elm	Early flowering obscures development of vegetative buds. BS1 occurs as flowers wither and fruits begin to develop
*Carya cordiformis* bitternut hickory	No bud scales so estimate transition from BS to BB
*Juglans nigra* black walnut	F2 leaf rachis is at full length but farthest leaflets not full size and take long time to finish; F3 all terminal leaflets at full size
*Celtis occidentalis* common hackberry	Early buds remain very small for some weeks. Do not count as swelling until appear conspicuously white. Swollen small white buds may include both leaves and flowers or only leaves; if only leaves, BS and BB are much later
*Gymnocladus dioicus* Kentucky coffeetree	Hard to see early BS; only BS3 conspicuous so back‐date to earlier week(s) for BS1 and BS2. Difficult to determine when tiny leaflets have reached F3
*Aesculus glabra* Ohio buckeye	Bottom branches start before canopy; use only canopy buds (but subjective where bottom stops). After BS3, rapid opening of scales to start BB and reveal pompom of leaves
*Carya laciniosa* shellbark hickory	Hard to judge anticipated final size and know when 1/3 of that size for BS1; declare BS1 when buds become conspicuous without binoculars
*Ulmus rubra* slippery elm	Early flowering obscures development of buds. BS1 occurs as flowers wither and fruits begin to develop
*Acer saccharum* sugar maple	Buds can have only leaves (with later BB) or leaves and flowers (with early BB). If much of canopy flowering, estimate BS and BB occur when flowers appear and elongate. If less canopy flowering, estimate BS only on leaf buds and BB on emerging leaves; ignore minor flowering
*Carpinus caroliniana* American hornbeam	Leaves elongate rapidly to near final size but much folding between veins. F3 coded when folding eliminated
*Asimina triloba* pawpaw	New leaves continue to emerge at end of elongating stem; F2 is non‐terminal leaves not full size; F3 = new leaves at end are full size. Elongated development depends on higher light
*Zanthoxylum americanum* common pricklyash	New leaves continue to emerge at end of elongating stem; F2 is non‐terminal leaves not full size; F3 = new leaves at end are full size. Elongated development depends on higher light
*Lindera benzoin* northern spicebush	New leaves continue to emerge at end of elongating stem; F2 is non‐terminal leaves not full size; F3 = new leaves at end are full size. Elongated development depends on higher light
*Toxicodendron radicans* eastern poison ivy	Hard to see BS1 and BS2; often estimated from BS3
*Vitis* spp. grape	Potential for continuing growth in summer so stopped coding at F2

Flowering Events	
Species	Issues and solutions
*Tilia americana* American basswood	Flowering observations in only 3 years; flowers late after F3 (and after all species in forest are at F3). Insect‐pollinated
*Carya cordiformis* bitternut hickory	Emerging stem/leaves obscure flowering so estimate Fl 1 as when BS begins
*Gymnocladus dioicus* Kentucky coffeetree	Flowering observations in only 5 years; flowers late after F3 (and after all species in forest are at F3). Insect, not wind‐pollinated
*Vitis* spp. grape	Flowering observations in only 8 years
*Parthenocissus quinquefolia* Virginia creeper	No observations of flowering

*Note:* See Table 4 for names of events abbreviated below.

**TABLE A3:** Description of criteria used to define leaf coloration phenological stage for each study species.

Species	Criteria
*Tilia americana* American basswood	Slow green to complete yellow before drop. In some years and in some individuals, top 1/4–1/3 of canopy turns color and can crinkle to brown while rest of canopy is green; coded as LC1 (1/3 of tree turned color)
*Ulmus americana* American em	Change to yellow; individuals quite different in when change
*Carya cordiformis* bitternut hickory	Change to yellow; some trees retain brown leaves for some weeks
*Juglans nigra* black walnut	Highly variable among ind. and yrs.; green to yellow before drop
*Fraxinus quadrangulata* blue ash	Late and indistinct change in color; becomes more dull green with little hint of yellow; can go from LC1 to LD3 in 1 week
*Quercus macrocarpa* bur oak	Slow change to dull yellow and into brown shades
*Celtis occidentalis* common hackberry	Green to pale green for very long time; sometimes some turn to yellow; can drop green; hard to distinguish LC1
*Fraxinus pennsylvanica* green ash	Change to yellow
*Gymnocladus dioicus* Kentucky coffeetree	Slow green to complete yellow before drop
*Quercus rubra* orthern red oak	Change to brown or in some years reddish‐brown. Some yrs. all drop at same time as other sp.; some ind. can drop quite late
*Aesculus glabra* Ohio buckeye	Bottom branches change color before canopy; use canopy dates (but subjective where bottom stops); some individuals become only brown; others yellow in some years; much variability among individuals; a few are very late each year; much variability among years
*Carya laciniosa* shellbark hickory	Some ind. in some yrs. change to yellow; others to brown; some ind. in some yrs. can drop brown leaves quite late; leaves persist as LC3/LD2 for some weeks; strong winds eventually remove all leaves = LD3
*Ulmus rubra* slippery elm	Most change from green to brown; some ind. have some yellow color
*Acer saccharum* sugar maple	Slow green to yellow or red/yellow; can be rapid in some yrs. In drought years, some ind. change to brown prematurely; never show other colors
*Fraxinus americana* white ash	Slow green to yellow or red/yellow; can be rapid in some years
*Carpinus caroliniana* American hornbeam	Change to yellow; can be quite rapid. If freeze before yellow, leaves persist as green/yellow for many weeks until blown off by high wind
*Asimina triloba* pawpaw	Highly variable; some ind. in some yrs. change to yellow; others retain green for long time, even with heavy frost damage, and drop green; can go from FE3 to LD3 in 1 week
*Zanthoxylum americanum* common pricklyash	Green to yellow before drop
*Lindera benzoin* northern spicebush	Slow green to yellow before complete drop
*Toxicodendron radicans* eastern poison ivy	Slow green to yellow with some red hues before drop
*Vitis* spp. grape	Slow green to yellow before complete drop
*Parthenocissus quinquefolia* Virginia creeper	Green to red/purple; can be very conspicuous

*Note:* Also included for some species are atypical criteria for defining Leaf Coloration and/or Leaf Drop stage. See Table 1 for names of events abbreviated below. Atypical weather effects by year are noted at bottom of table. Additional notes of atypical weather effects by year: 1995, 1998, 2000 some individuals' leaves freeze; 1996 leaves look dry; 1997 autumn events very late; 1999 drought common effect at top of American basswood; 2012 all Ohio buckeye very early; 2013 common hackberry and sugar maple leaves show drought; 2019 hackberry, pawpaw, Amer. basswood, Amer. hopbeam leaves freeze while green.

**TABLE A4:** Equations used to calculate durations of phenological stages.

Stage	Events defining duration	Equation
Bud Swell	Bud Swell 1 − Bud Burst 1	BB1 − BS1
Bud Burst/Leafing Out	Bud Burst 1 − Leaf Expansion 1	LE1 − BB1
Leaf Expansion	Leaf Expansion 1 − Leaf Expansion 3	LE3 − LE1
Flowering	Flowering 1 − Flowering 2	FL2 − FL1
Leaf Coloration	Leaf Coloration 1 − Leaf Coloration 3	LC3 − LC1
Leaf Drop	Leaf Drop 1 − Leaf Drop 3	LD3 − LD1
Spring Phenology	Bud Swell 1 − Leaf Expansion 3	LE3 − BS1
Autumn Phenology	Leaf Coloration 1 − Leaf Drop 3	LD3 − LC1
Growing Season^a^	Leaf Expansion 1 − Leaf Coloration 1	LC1 − LE1

*Note:* Day of year of two phenological events delimit each phenological stage.

^a^ Estimated period of photosynthetic activity.

**TABLE A5:** Species nomenclature, common name, growth form, and seasonal group classifications used in analyses.

Species^a^	Common Name; Growth Form	Abbr	Begin Bud Swell	Begin Bud Burst	Begin Leaf Expansion^b^	End Leaf Expansion	Begin Flowering	Begin Leaf Coloration	End Leaf Coloration	Begin Leaf Drop	End Leaf Drop
*Tilia americana* L	Amer. basswood; canopy tree	AB	Late	Late	Late	Intermed	N/A	Intermed	Late	Intermed	Intermed
*Ulmus americana* L.	American elm; canopy tree	AE	Late	Late	Late	Intermed	Early	Late	Late	Late	Late
*Carya cordiformis* (Wangenh.) K. Koch	bitternut hickory; canopy tree	BH	Late	Late	Late	Intermed	Late	Late	Late	Late	Late
*Juglans nigra* L.	black walnut; canopy tree	BW	Late	Late	Late	Late	Late	Intermed	Intermed	Intermed	Intermed
*Fraxinus quadrangulata* Michx.	blue ash; canopy tree	BA	Late	Late	Late	Intermed	Late	Late	Late	Late	Late
*Quercus macrocarpa* Michx.	bur oak; canopy tree	BO	Late	Late	Late	Intermed	Late	Late	Late	Late	Late
*Celtis occidentalis* L	common hackberry; canopy tree	HB	Late	Late	Late	Intermed	Late	Late	Late	Late	Late
*Fraxinus pennsylvanica* Marshall	green ash; canopy tree	GA	Late	Late	Late	Intermed	Late	Intermed	Intermed	Intermed	Intermed
*Gymnocladus dioicus* (L.) K. Koch	Kentucky coffeetree; canopy tree	KC	Late	Late	Late	Late	N/A	Intermed	Intermed	Intermed	Intermed
*Quercus rubra* L.	northern red oak; canopy tree	RO	Late	Late	Late	Intermed	Late	Late	Late	Late	Late
*Aesculus glabra* Willd.	Ohio buckeye; canopy tree	OB	Late	Late	Early	Early	Late	Early 2	Early 2	Early 2	Early 2
*Carya laciniosa* (Michx. f.) G. Don	shellbark hickory; canopy tree	SH	Late	Late	Late	Intermed	Late	Late	Intermed	Intermed	Late
*Ulmus rubra* Muhl.	slippery elm; canopy tree	SE	Late	Late	Late	Intermed	Early	Late	Late	Intermed	Late
*Acer saccharum* Marshall	sugar maple; canopy tree	SM	Late	Late	Late	Intermed	Late	Late	Late	Late	Late
*Fraxinus americana* L.	white ash; canopy tree	WA	Late	Late	Late	Intermed	Late	Intermed	Intermed	Intermed	Intermed
*Carpinus caroliniana* Walter	Amer. hornbeam; treelet	ah	Late	Late	Late	Intermed	Late	Late	Late	Intermed	Intermed
*Asimina triloba* (L.) Dunal	pawpaw; treelet	pp	Late	Late	Late	Late	Late	Late	Late	Late	Late
*Zanthoxylum americanum* Mill.	common pricklyash; shrub	pa	Late	Late	Late	Late	Late	Late	Late	Late	Late
*Lindera benzoin* (L.) Blume	northern spicebush; shrub	sb	Late	Late	Late	Intermed	Early	Late	Intermed	Intermed	Intermed
*Toxicodendron radicans* (L.) Kuntze	eastern poison ivy; vine	pi	Late	Late	Late	Intermed	N/A	Intermed	Intermed	Intermed	Intermed
*Vitis* spp. L.	grape species mixture; vine	gr	Late	Late	Late	N/A	N/A	Intermed	Intermed	Intermed	Intermed
*Parthenocissus quinquefolia* (L.) Planch	Virginia creeper; vine	vc	Late	Late	Late	Intermed	N/A	Intermed	Intermed	Intermed	Intermed
*Fraxinus quadrangulata* Michx.	blue ash; sapling	ba	Late	Late	Late	Intermed	N/A	Late	Late	Late	Late
*Aesculus glabra* Willd.	Ohio buckeye; sapling	ob	Early	Early	Early	Early	N/A	Early 1	Early 1	Early 1	Early 1
*Acer saccharum* Marshall	sugar maple; sapling	sm	Late	Late	Late	Intermed	N/A	Late	Late	Late	Late

*Note:* Abbreviation of species' common names are used for visualization in figures.

^a^ Species nomenclature follows USDA, NRCS. 2024. The PLANTS Database (http://plants.usda.gov) 22 January 2024. National Plant Data Team, Greensboro, NC 27401‐4901 USA.

^b^ For durations, each species stayed in the same seasonal group as the event marking the beginning of a phenophase, including species in different groups for beginning and end of a phenophase (e.g., *Tilia americana* Leaf Coloration Duration is intermediate).

**TABLE A6:** Weather variables defined.

Name	Description
Temperature	
Mean daily mean temperature (*T*)	Σ (*T*_mean_)/(# days in time interval)
Mean minimum temperature (*T*_min_)	Σ (*T*_daily‐min_)/(# days in time interval)
Growing degree day (GDD)	Σ (*T*_mean_ − *T*_b_); if *T*_mean_ < *T*_b_, GDD = 0^a^
Chilling degree day (CDD)	Σ (*T*_b_ − *T*_mean_); if *T*_mean_ > *T*_b_, CDD = 0^a^
Chilling degree day_i_ (CDD_i_)	Σ (*T*_b_ − *T*_min_); if *T*_daily‐min_ > *T*_0°C_, CDD_i_ = 0^b^
Hot days (HD)	Number of days with *T* ≥ 32.2°C
Frost days (FD)	Number of days with ≤ 0°C
Precipitation	
Total precipitation (P)	Total precipitation (cm)
Rain days (RD)	Number days with precipitation ≥ 2.5 mm
Flood days (FLD)	Number days with precipitation ≥ 20 mm
Drought weeks (DR)	Number weeks of 7 consecutive days with no precipitation

*Note:* For GDD and CDD, subscripts (e.g., GDD_X_) indicate the base temperature (*T*_b_) in °C. See Appendix A: Tables A7a, A7b for details on base temperatures used.

^a^ *T*_mean_: Daily mean temperature, [(daily max *T* + daily min *T*)/2]; *T*_b_: Base temperature.

^b^ *T*_daily‐min_: Daily minimum temperature; *T*_b_: Base temperature.

**TABLE A7a:** For each combination of event‐seasonal group, the range (across species) of mean day of year and mean date of event, and variables (weather variables and legacy events) used to predict spring phenology.

Spring	Weather variable						Legacy event
Event Seasonal group Range of DOY Date(s) of event	DOY‐Stop Date	Temp.	GDD^a^	CDD^b^	Precip. total rain days flood days	Previous year temp. and precip	
Begin Bud Swell Ohio Buckeye sapling 81 22 Mar	80 21 Mar	6–3	6–3	6–3	4–2	Summer (Jun–Aug) Autumn (Sep–Nov)	
Begin Bud Swell Late 90–110 31 Mar–20 Apr	99 9 Apr	6–3	6–3	6–3	4–2	Summer (Jun–Aug) Autumn (Sep–Nov)	
Begin Bud Burst Ohio Buckeye Sapling 91 Apr 1	90 31 Mar	4–2	4–2	4–2	4–2	Summer (Jun–Aug) Autumn (Sep–Nov)	Begin Bud Swell
Begin Bud Burst Late 100–120 10–30 Apr	109 19 Apr	4–2	4–2	4–2	4–2	Summer (Jun–Aug) Autumn (Sep–Nov)	Begin Bud Swell
Begin Leaf Expand Ohio Buckeye sapling and tree 100–107; 10–17 Apr	103 13 Apr	4–2	4–2		4–2	Summer (Jun–Aug) Autumn (Sep–Nov)	Begin Bud Swell
Begin Leaf Expand Ohio Buckeye sapling & tree 100–107; 10–17 Apr	103 13 Apr	4–2	4–2		4–2	Summer (Jun–Aug) Autumn (Sep–Nov)	Begin Bud Swell
Begin Leaf Expand Late 117–134 27 Apr–May 14	125 5 May	4–2	4–2		4–2	Summer (Jun–Aug) Autumn (Sep–Nov)	Begin Bud Swell Begin Bud Burst
End Leaf Expand Ohio Buckeye sapling and tree 114–118; 24–28 Apr	115 25 Apr	4–2	4–2		4–2		Begin Bud Swell Begin Bud Burst Begin Leaf Expansion
End Leaf Expand Intermediate 128–142 8–22 Ma	134 14 May	4–2	4–2		4–2		Begin Bud Swell Begin Bud Burst Begin Leaf Expansion
End Leaf Expand Late 148–159 28 May–8 Jun	153 2 Jun	4–2	4–2		4–2		Begin Bud Swell Begin Bud Burst Begin Leaf Expansion
Begin Flowering Early 88–96 29 Mar–6 Apr	91 1 Apr	6–3	6–3	6–3	4–2	Summer (Jun–Aug) Autumn (Sep–Nov)	Begin Bud Swell Begin Bud Burst

*Note:* Also, for each weather variable, Stop Date after which no weather values are used and number of 30‐day and 60‐day intervals of weather data used, starting from Stop Date back to earlier months. For example, for “6–3” of Temp. for Begin Bud Swell of Ohio buckeye saplings, mean daily temperatures are used from DOY 80 (Stop Date) of current year back to DOY 264 of the prior year at six monthly (m) 30‐day intervals and three seasonal (s) 60‐day intervals. For weather variables from the previous year, the same three‐month time periods were used for summer and autumn, regardless of the mean date of the event. For temperature (*T* and *T*_min_), the mean across the 30‐day months or 60‐day seasons are calculated. For all other variables, the sum across the months or seasons is calculated. See Appendix A: Table A6 for description of each weather variable.

^a^ GDD: used multiple base temperatures in 2°C intervals from 0°C to 12°C; for example, GDD4 = growing degree days with base temperature of 4°C.

^b^ CDD: used multiple base temperatures in 2°C intervals from −4°C to 8°C; for example, CDD4 = chilling degree days with base temperature of 4°C.

**TABLE A7b:** For each combination of event‐seasonal group, the range (across species) of mean day of year and mean date of the event, and variables (weather variables and legacy events) used to predict autumn phenology.

Autumn	Weather variable						Legacy event
Event Seasonal group Range of DOY Date(s) of event	DOY‐Stop Date	Temp.	*T*_min_ ^a^	CDD^b^ CDD_i_ ^c^ flood days	Hot days	Precip. total rain days drought weeks	
Begin Leaf Color Ohio Buckeye sapling 195 14 Jul	194 13 July	6–3			4–2	4–2	End Leaf Expansion
Begin Leaf Color Ohio Buckeye tree 244 1 Sep	243 31 Aug	6–3			4–2	4–2	End Leaf Expansion
Begin Leaf Color Intermediate 269–282 26 Sep–9 Oct	275 2 Oct	6–3	6–3	2–1	4–2	4–2	End Leaf Expansion
Begin Leaf Color Late 285–298 12–25 Oct	291 18 Oct	6–3	6–3	2–1	4–2	4–2	End Leaf Expansion
Begin Leaf Drop Ohio Buckeye sapling 209 28 Jul	208 27 Jul	6–3			4–2	4–2	End Leaf Expansion Begin Leaf Coloration
Begin Leaf Drop Ohio Buckeye tree 256 13 Sep	255 12 Sep	6–3			4–2	4–2	End Leaf Expansion Begin Leaf Coloration
Begin Leaf Drop Intermediate 283–300 10–27 Oct	291 18 Oct	6–3	6–3	2–1	4–2	4–2	End Leaf Expansion Begin Leaf Coloration
Begin Leaf Drop Late 301–316 28 Oct–12 Nov	308 4 Nov	6–3	6–3	2–1	4–2	4–2	End Leaf Expansion Begin Leaf Coloration

*Note:* See Appendix A: Table 7a A7a for other details.

^a^ *T*_min_: daily minimum temperature.

^b^ CDD: used multiple base temperatures in 2°C intervals from −4°C to 8°C; for example, CDD4 = chilling degree days with base temperature of 4°C.

^c^ CDD_i_ calculated from daily minimum temperature and the same intervals as CDD.

## Appendix B

### Text 1. Description of Phenological Events and Stages

#### Spring Vegetative Stages

Dates of three “events” within each of three vegetative “stages” are documented. Each “stage” has a beginning date, intermediate date, and ending date, each date a so‐called phenological “event” of the beginning of a “substage.” Only beginning and ending dates were used in analyses; intermediate dates were useful for better estimation of unobserved events.

*Bud Swelling (BS)*: a bud expands in size and reveals underlying parts of bud scales, in most species changing to new, more conspicuous colors, such as green or reddish brown/rust. Green may show beyond brown scales at very tip of bud, but no emergence of shoot/leaves occurs yet. The first event of Bud Swelling, BS1, is usually visible from ground level without binoculars (for most species when buds are 1/3 of final size). Subsequent events indicate a bud has grown to 2/3 (BS2) and final size (BS3).

Bud Burst (Shoot/Leaf Emergence; Leaf Out) (BB): a bud opens and shoots/leaves begin growth. In the first event of Bud Burst (BB1), green shoot/leaf parts become visible as they extend beyond the end of bud scales. Subsequent events indicate when 2/3 of the leaf/shoot emergence from bud scales occurs but without leaf unfolding or stem elongation (BB2), and ends when leaf petioles are fully visible and the leaf is unfolded, the entire blade fully visible but without substantive expansion (BB3).

*Leaf Expansion (LE)*: begins when the unfolded leaf blade is 1/3 of its final length (LE1), continues to 2/3 of its final length (LE2), and ends at full leaf expansion (LE3). LE3 leaves are not “hardened” or their final deep green color.

*Leaf Maturity (LM)*: an event only; the date when leaves have ‘hardened’ and have final deep green color. Five years of observations (1995–1999) indicate that maturation is complete about 1–2 weeks after LE3 (among 21 species, mean = 9.5 days; range = 8.4–11.5 days) (see Dryas Archived Data Set 1). Descriptive statistics are calculated, but no analyses use this metric.

#### Flowering Stage

Flowering begins when 30% flowers are full size and appear functional (FL1) and ends 1 week after > 70% of flowers appear non‐functional (dried up/petals wilted) (FL2). For most canopy tree species, the period with a functional pistil or shedding of pollen is not easily observed. Duration of functional flowers may differ from the duration of observed phenology (End—Begin Flowering). All but three species (see below) are wind‐pollinated. Criteria to define flowering is species‐specific and dependent on the type of inflorescence, as follows:

Catkin‐bearing species:

- Begin Flowering date: catkin fully elongated;
- End Flowering date: catkin dried up.

*Fraxinus* species:

- Begin Flowering date: inflorescence expands to its global full size;
- End Flowering date: inflorescence dried up.

*Ulmus* species with highly visible stamens:

- Begin Flowering: yellow elongated stamens;
- End Flowering: brown stamens.

Insect‐pollinated species: *Aesculus glabra, Tilia americana*, and *Gymnocladus dioicus* :

- Begin Flowering: flower petals at maximum size;
- End Flowering: flower petals beginning to wilt and/or change color.

#### Autumn Vegetative Stages

Dates of three events within each of two vegetative stages are documented.

*Leaf Coloration (LC)*: Leaves convert their green color to the senescent color. Events of LC1, LC2, and LC3 are when 1/3, 2/3 and total conversion occurs (see below).

*Leaf Drop (LD)*: LD1, LD2, and LD3 represent 1/3, 2/3, and all leaves drop.

Phenological changes are often not highly synchronous within the large canopy crowns. For events of BS1, BB1, LE1, LC1, and LD1, synchrony within the crown was assumed, that is, a judgment of the extent of phenological development was integrated across the entire crown to represent the dominant state; (e.g., for BS1, BB1, LE1, LC1, and LD1 about 1/3 (25%–45%) of the vegetative units display the event state; for BS2, BB2, LE2, LC2, and LD2 about 2/3 (55%–80%) of units develop to the event state, and for BS3, BB3, LE3, LC3 and LD3 (95%–100%) of units are at the final state). In some species, for example, *Tilia americana* , development in spring and senescence in autumn begins at the canopy top and moves downward. In *Aesculus glabra* , both seasons' changes begin on short, secondary, low branches, and then move upward into the crown. See individual species descriptions in Appendix S1: Tables S1, S2 for details of these general criteria.

Autumn phenological stages are problematic because (1) changes may not be synchronous within the crown, (2) leaf coloration varies both with rate of conversion within a leaf and % of leaves at a given conversion state, and (3) the two stages are often non‐sequential. Thirteen contrasting sequences combining Leaf Coloration and Leaf Drop events were observed. A date represents an attempt to integrate this variation and judge the dominant color of the crown (i.e., the dominant color is LC1 = 1/3 of green converted to final color, LC2 = 2/3 of green converted, and LC3 = all green gone and leaves are at final senescent color). Dates of leaf coloration represents only the leaves remaining on the individual. See individual species descriptions in Appendix S1: Table S3 for details of these general criteria.

#### Duration of Stages and Seasons

Duration of stages was calculated from the first event of the stage until the first event of the next stage for Bud Swell and Bud Burst. For all other stages, it was calculated from the first event to the last event of the stage (Appendix S1: Table S4). Three seasonal durations also were calculated from event dates: (1) Spring Phenology = (Leaf Expansion 3—Bud Swell 1), (2) Autumn Phenology = (Leaf Drop 3—Leaf Coloration 1), and (3) Growing season = (Leaf Coloration 1—Leaf Expansion 1). These latter two events for the growing season define the estimated period of photosynthetic activity.

### Text 2. Details of Statistical Analysis

Analyses for (a) temporal change in event start date, (b) temporal change of stage duration, and (c) weather and/or legacy variables that best predicted event date were conducted using linear mixed‐effects models. All analyses were based on event dates observed on individual plants in each year, with stage durations calculated from these event dates. Analyses were structured to differentiate species and different life forms within a species (for three species, data from both canopy trees and saplings were collected: *Acer saccharum, Fraxinus quadrangulata*, and *Aesculus glabra* ). Statistical methods were based on the methods of Xie et al. (2018). All mixed‐effects models were conducted using R version 4.0.4 (R Core Team 2021) and the *lme4* package (Bates et al. 2015).

#### Statistical Analysis of Event Dates and Stage Durations

Nine stages were analyzed to test for temporal change in event dates, five stages in spring and four stages in autumn (Appendix B: Text 1; Appendix C: Tables C1a and C1b). For each, separate analyses were conducted for groups of species that had similar timing (Appendix A: Table A5; Appendix C: Tables C1a and C1b). Species were separated into 4 groups for each phenological event for autumn. A separate set of mixed‐effects models was created for each event‐season combination. Observations from individual plants were used in analyses. The response variable was the event date, measured as day of year. All models included random intercepts for individual plants. When a seasonal group had more than one species, all models also included a random intercept for species (canopy trees and saplings within a single species were treated as separate groups). The fixed effects structure and random effects structure were selected in separate steps using different information criteria.

First, the fixed effects structure was chosen. The Akaike information criterion corrected for sample size (AIC_C_) was used to compare a model that included year as a predictor of event date (where the start year, 1993, was *year* = 0), and a null model without year. When there were more than three species, both models in this initial step included random slopes for year (uncorrelated with the random intercept) assigned to each species. In seasonal groups with two or three species, the species were assigned random intercepts only. In seasonal groups with only one species, species was excluded from the random effects structure (only random intercepts for individual plant were included).

Second, after testing the optimal fixed effects structure, the optimal random effects structure was selected using conditional AIC (cAIC) (Greven and Kneib 2010). For seasonal groups with more than three species, three models with different random effects structures were compared using the cAIC. Each of the three models included the fixed effects structure chosen in the previous step. The three competing random effects structures were: (1) each species has a random slope and intercept that are uncorrelated, (2) each species has a random slope and intercept that are correlated, and (3) each species has a random intercept only (no random slopes). The *cAIC4* package (Saefken and Ruegamer 2018; Saefken et al. 2018) was used to calculate cAIC values. Model comparisons with cAIC were not completed for seasonal groups with three or fewer species. Seasonal groups with two or three species included only random intercepts for species, and seasonal groups with only one species had only individual plant in the random effects structure.

The temporal changes in the durations of nine stages through the 29 years of this study were also tested (five in spring, three in autumn, and Growing Season spanned the two seasons; Appendix A: Table A4; Appendix C: Tables C4a and C4b). Species were separated into seasonal groups with similar phenology, based on the first event marking the stage. Each stage had 2–4 seasonal groups with 24 combinations of stage and group. Separate analyses were conducted for each combination of stage and seasonal group. The same analytical approach was used for the durations of stages as described for event dates, except that the response variable was stage duration measured in days.

To test the influence of higher phylogenetic effects, we also constructed a set of temporal change models with genus, family, and order in the random effects structure. Each additional phylogenetic level was included separately, with species nested within each. As an initial investigation, we tested the influence of the higher phylogenetic levels for three response variables, one event from each season and one duration: Begin Leaf Expansion, Begin Leaf Coloration, and Growing Season Duration. We found no improvement of model performance for Begin Leaf Coloration in intermediate species, and marginal improvement (∆AIC_C_ between 0.06 and 0.36) for the other seasonal groups and response variables. Thus, we proceeded with models that included only species in the random effects structure and do not report results for the higher phylogenetic categorizations.

#### Statistical Analysis of Weather Variables Predicting Event Dates

For analyses of the relationship between event dates and weather variables, the dates of seven phenological events were tested in 19 separate analyses based on seasonal groups of species (Appendix A: Table A5). These events and seasonal groups were the same as for the analyses of the temporal trend in event dates, except that End Leaf Coloration and End Leaf Drop were excluded. These two variables were excluded because the dates on which they occur were largely constrained and determined by the preceding event date, Begin Leaf Coloration or Begin Leaf Drop.

In all analyses, the response variable was the event date, measured as day of year. Fixed effects consisted of weather variables and, in most cases, phenological variables that preceded the response (legacy effects). Weather variables described temperature and precipitation in multiple 30‐day intervals (“months”) or 60‐day intervals (“seasons”) preceding the typical date for an event within a seasonal group (see Appendix A: Tables A7a and A7b for details on which weather variables predicted specific event dates). Standardized values of explanatory variables (both weather variables and legacy effects), scaled to have a mean of zero and standard deviation of one, were used in all analyses. However, raw (unscaled) values were used for Tables, except in part of Appendix C: Table C6.

The optimal fixed effects and random effects structures were chosen for each weather or legacy variable individually, in a set of models where the variable was used to predict the date of the event in question. Three main steps were completed to select weather variables that most strongly predicted a phenological event:

1. For each time period (month or season) of a weather variable, AICc was used to compare the fit of models that included (a) the weather variable as a fixed effect with uncorrelated random species slopes and intercepts, (b) the same fixed effect and a random species intercept (no random slope), (c) no fixed effect with uncorrelated random species slope and intercept, and (d) no fixed effect with a random species intercept. For growing degree days (GDD), chilling degree days (CDD), and chilling degree days_i_ (CDD_i_), step 1 was preceded by an investigation of which temperature threshold most strongly predicted the response variable. The thresholds tested for GDD ranged from 0°C to 12°C, in 2°C intervals. The thresholds tested for CDD and CDD_i_ ranged from −4°C to 8°C, in 2°C intervals. If either model with no fixed effect had the best fit, that is, (c) or (d) above, that particular weather variable was excluded from further steps. In all cases, random intercepts for individual plants were included. In seasonal groups with two or three species, only models with random species intercepts (no random slopes) were created, thus only models (b) and (d) were compared. For seasonal groups with only one species, species was not included in the random effects, and models (b) and (d) were compared. All models at this stage were constructed using maximum likelihood.
2. Steps were taken to prevent multicollinearity in the models. Pairs of highly correlated weather variables were determined (|*r*| > 0.5). When weather variables were correlated, variables whose models had inferior (greater) AIC_C_ scores were excluded. This left a list of legacy variables and uncorrelated weather variables as potential fixed effects, each of which was associated with a random species intercept or a random slope plus intercept.
3. Models that had different combinations of the uncorrelated fixed effects were compared. The candidate models were limited to those that had three or fewer fixed effects because of computational limitations. The five top models with respect to AIC_C_ are presented (Appendix C: Tables C5a and C5b). The most influential fixed effects were determined by how often they were included in the top models, as well as by *t*‐values. Fixed effects included in the top model and at least four of the top five models were highlighted as top explanatory variables. When multiple variables met these criteria, the one with the greatest absolute *t*‐value in the top model was treated as the most important fixed effect.

For the weather variables chosen as important explanatory variables of a specific event date, we used linear regression to test for temporal change in the weather variable through time (1993–2021). After we found the best model of weather and legacy variables predicting an event date, we repeated the tests of temporal change for the event date (linear mixed‐effects model) and weather variable (linear regression) for a shorter time period, 1993–2017. We ran this second set of analyses because of high variability of weather from 2018 to 2021.

#### Overall

In all three sets of analyses (temporal changes of event dates, temporal changes of stage duration, and weather or legacy variables that predict event dates), models were initially constructed using maximum likelihood for comparison with information criteria. Once the preferred model was chosen, final model parameters were determined using restricted maximum likelihood. Confidence intervals for the species‐level coefficients of the favored model were estimated using 10,000 simulations with the *arm* package (Gelman and Su 2020), using posterior simulations as described by Gelman and Hill (2007). Two approaches were used to evaluate models; see Appendix B: Text 3 for details.

### Text 3. Model Evaluation and Validation

#### Methods

The top mixed‐effects models were obtained for tests of: (a) temporal shifts in dates of phenological events (27 total models, each representing an event‐season combination), (b) temporal shifts in duration of stages (24 stage–season combinations), and (c) weather predictors for phenological events (19 event–season combinations). Then the fits of these linear mixed‐effects models were evaluated in two ways (Appendix D: Figures D1, D2, D3, D4). First, the *R* ^*2*^ was calculated for the relationship between the predicted values from the model and observed values in the data. Second, 10‐fold cross‐validation was used to calculate the root mean square error (RMSE) for each model, with one replicate. Within each analysis, one‐tenth of the data were randomly selected as the test set, while the remaining nine‐tenths of the data were used to train the model. The selection of a different one‐tenth of the data as a test set was repeated until every data point was used in the test set (10 total times). The RMSE was calculated as the mean across the 10 tests. A simple random sample design was used, without consideration of grouping factors. This approach was modeled after Xie et al. (2018).

#### Results

In model evaluations, 10‐fold cross‐validations of the nine events (27 events–seasonal group combinations) showed that model prediction uncertainties averaged 9.6 days (range 7.4–18.2, median 8.4), as measured by the root‐mean‐square error (RMSE). Correlations between predicted and observed dates yielded *R* ^2^ values that averaged 0.30 (range 0.02–0.49, median 0.37; Appendix D: Figures D1 and D2). For nine durations (24 duration–seasonal group combinations), model prediction uncertainties averaged 7.8 days (range 3.8–13.1, median 7.2), and *R* ^2^ of the correlation between observed dates and model predictions averaged 0.26 (range 0.01–0.77, median 0.24; Appendix D: Figure D3).

In evaluating the 19 models with weather variables for spring and autumn (see Appendix B: Text 3), 10‐fold cross‐validations showed that model prediction uncertainties averaged 5.8 days (range 2.8–11.7, median 5.2) and *R* ^2^ averaged 0.71 (range 0.40–0.89, median 0.76; Appendix D: Figure D4).

### Text 4. Random Effects Variance in Statistical Models

In spring, for all model types, species intercept dominated random effect structure (Appendix C: Table C10a). In 11 models that tested for changes in event dates through time, most variation was observed in species rather than individual plant intercepts. The SD of the individual intercept was at most 48.6% of the SD of the species intercept (mean 30.3%, median 32.0%); SD across all individuals never exceeded 2.0 days. In the models testing weather variables that predict phenological events, similar to Xie et al. (2018), random intercepts of species had greater standard deviations than weather variables' random slopes, with a 2‐ to 11‐fold difference (Appendix C: Table C10a).

In autumn, in three of four model types that tested for changes in event dates through time, more variation was observed in species than individual intercepts; for Begin Leaf Coloration of the Intermediate group, SD of species and individual intercepts were essentially the same (Appendix C: Table C10b).

In models testing weather variables that predict phenological events, random effects of species had greater standard deviations than weather variables, with a 2‐ to 19‐fold difference (Appendix C: Table C10b).

## Appendix C

**TABLE C1a:** Model selection for temporal change in each of five spring phenological events for seasonal groups of species (and growth forms) in Trelease Woods (1993–2021).

Event—Seasonal group	*N*	Fixed effects				Random effects			
		Model	df	ΔAICc	Akaike weight	Model	Fixed effect	df	ΔcAIC
BEGIN BUD SWELL‐Early Ohio buckeye sapling	1	Year as fixed effect	4	0	0.921	Correlated^a^	N/A	N/A	N/A
		No fixed effect	3	4.90	0.079	Uncorrelated^b^	N/A	N/A	N/A
						Random intercept	N/A	N/A	N/A
BEGIN BUD SWELL‐ Late	24	Year as fixed effect	6	0	0.987	Correlated^a^	Year	136.3	0
		No fixed effect	5	8.74	0.013	Uncorrelated^b^	Year	136.9	1.14
						Random intercept	Year	124.9	103.14
BEGIN BUD BURST‐Early Ohio Buckeye sapling	1	Year as fixed effect	4	2.02	0.267	Correlated^a^	N/A	N/A	N/A
		No fixed effect	3	0	0.733	Uncorrelated^b^	N/A	N/A	N/A
						Random intercept	N/A	N/A	N/A
BEGIN BUD BURST‐Late	24	Year as fixed effect	6	0	1.000	Correlated^a^	Year	180.0	0
		No fixed effect	5	20.26	0.000	Uncorrelated^b^	Year	180.3	1.37
						Random intercept	Year	166.5	76.46
BEGIN LEAF EXPANSION‐Early Ohio buckeye sapling + tree	2	Year as fixed effect	5	0.94	0.384	Correlated^a^	N/A	N/A	N/A
		No fixed effect	4	0	0.616	Uncorrelated^b^	N/A	N/A	N/A
						Random intercept	N/A	N/A	N/A
BEGIN LEAF EXPANSION‐Late	23	Year as fixed effect	6	0	1.000	Correlated^a^	Year	173.5	0.35
		No fixed effect	5	18.40	0.000	Uncorrelated^b^	Year	173.3	0
						Random intercept	Year	155.0	125.18
END LEAF EXPANSION‐Early Ohio buckeye sapling + tree	2	Year as fixed effect	5	0	0.467	Correlated^a^	N/A	N/A	N/A
		No fixed effect	4	0.26	0.533	Uncorrelated^b^	N/A	N/A	N/A
						Random intercept	N/A	N/A	N/A
END LEAF EXPANSION‐Intermediate	18	Year as fixed effect	6	0	0.999	Correlated^a^	Year	123.0	0
		No fixed effect	5	15.17	0.001	Uncorrelated^b^	Year	122.5	1.28
						Random intercept	Year	109.6	71.71
END LEAF EXPANSION‐Late	4	Year as fixed effect	6	0	0.601	Correlated^a^	Year	46.3	0.38
		No fixed effect	5	0.82	0.399	Uncorrelated^b^	Year	46.2	0
						Random intercept	Year	39.0	74.21
BEGIN FLOWERING‐Early	3	Year as fixed effect	5	0	0.999	Correlated^a^	N/A	N/A	N/A
		No fixed effect	4	13.56	0.001	Uncorrelated^b^	N/A	N/A	N/A
						Random intercept	N/A	N/A	N/A
BEGIN FLOWERING‐Late	14	Year as fixed effect	6	0	0.989	Correlated^a^	Year	58.5	0.5
		No fixed effect	5	9.04	0.011	Uncorrelated^b^	Year	57.6	0
						Random intercept	Year	51.3	3.09

*Note:* Model selection was conducted in two steps. Step 1 evaluated year as a fixed effect; year was used as a predictor if its model AICc value was less than that of the null model. Step 2 evaluated the structure of random effects, using the fixed effects structure from Step 1. Step 2 was not done when there were fewer than four species because random slopes could not be tested, and the model with random intercept was immediately chosen after Step 1. The model with the lowest conditional AIC (cAIC) was considered the top model. *N* represents the number of species (counting saplings and canopy trees separately for three species) in each analysis. 1 = correlated random slope and intercept; 2 = uncorrelated random slope and intercept.

^a^ Correlated random slope and intercept.

^b^ Uncorrelated random slope and intercept.

**TABLE C1b:** Model selection for temporal change in each of four autumn phenological events for seasonal groups of species (and growth forms) in Trelease Woods (1993–2021).

Event—Seasonal group	N	Fixed Effects				Random Effects			
		Model	df	ΔAICc	Akaike weight	Model	Fixed effect	df	ΔcAIC
BEGIN LEAF COLORATION‐ Early Ohio buckeye sapling	1	Year as fixed effect	4	1.48	0.323	Correlated^a^	N/A	N/A	N/A
		No fixed effect	3	0	0.677	Uncorrelated^b^	N/A	N/A	N/A
						Random intercept	N/A	N/A	N/A
BEGIN LEAF COLORATION‐ Early Ohio buckeye tree	1	Year as fixed effect	4	0	0.829	Correlated^a^	N/A	N/A	N/A
		No fixed effect	3	3.15	0.171	Uncorrelated^b^	N/A	N/A	N/A
						Random intercept	N/A	N/A	N/A
BEGIN LEAF COLORATION‐Intermediate	8	Year as fixed effect	6	0	0.990	Correlated^a^	year	97.3	0.94
		No fixed effect	5	9.8	0.100	Uncorrelated^b^	year	97.3	0
						Random intercept	year	91.4	52.25
BEGIN LEAF COLORATION‐Late	15	Year as fixed effect	6	0	1.000	Correlated^a^	year	167.0	0.35
		No fixed effect	5	20.40	0.000	Uncorrelated^b^	year	166.7	0
						Random intercept	year	153.5	146.16
END LEAF COLORATION‐Early Ohio buckeye sapling	1	Year as fixed effect	4	0.75	0.408	Correlated^a^	N/A	N/A	N/A
		No fixed effect	3	0	0.592	Uncorrelated^b^	N/A	N/A	N/A
						Random intercept	N/A	N/A	N/A
END LEAF COLORATION‐Early Ohio buckeye tree	1	Year as fixed effect	4	1.16	0.359	Correlated^a^	N/A	N/A	N/A
		No fixed effect	3	0	0.641	Uncorrelated^b^	N/A	N/A	N/A
						Random intercept	N/A	N/A	N/A
END LEAF COLORATION‐Intermediate	9	Year as fixed effect	6	0	0.990	Correlated^a^	year	100.7	0.38
		No fixed effect	5	9.18	0.010	Uncorrelated^b^	year	100.4	0
						Random intercept	year	93.9	41.58
END LEAF COLORATION‐Late	14	Year as fixed effect	6	0	1.000	Correlated^a^	year	159.2	0
		No fixed effect	5	27.91	0.000	Uncorrelated^b^	year	158.7	0.19
						Random intercept	year	148.9	37.67
BEGIN LEAF DROP‐Early Ohio buckeye sapling	1	Year as fixed effect	4	0.39	0.451	Correlated^a^	N/A	N/A	N/A
		No fixed effect	3	0	0.549	Uncorrelated^b^	N/A	N/A	N/A
						Random intercept	N/A	N/A	N/A
BEGIN LEAF DROP‐Early Ohio buckeye tree	1	Year as fixed effect	4	0	1.000	Correlated^a^	N/A	N/A	N/A
		No fixed effect	3	53.02	0.000	Uncorrelated^b^	N/A	N/A	N/A
						Random intercept	N/A	N/A	N/A
BEGIN LEAF DROP‐Intermediate	12	Year as fixed effect	6	0	0.772	Correlated^a^	year	151.4	0.64
		No fixed effect	5	2.43	0.228	Uncorrelated^b^	year	151.5	0
						Random intercept	year	141.3	84.06
BEGIN LEAF DROP‐Late	11	Year as fixed effect	6	0	0.907	Correlated^a^	year	120.4	11.66
		No fixed effect	5	4.54	0.093	Uncorrelated^b^	year	116.6	0
						Random intercept	year	107.8	96.55
END LEAF DROP‐Early Ohio buckeye sapling	1	Year as fixed effect	4	0	0.667	Correlated^a^	N/A	N/A	N/A
		No fixed effect	3	1.39	0.333	Uncorrelated^b^	N/A	N/A	N/A
						Random intercept	N/A	N/A	N/A
END LEAF DROP‐Early Ohio buckeye tree	1	Year as fixed effect	4	0	0.900	Correlated^a^	N/A	N/A	N/A
		No fixed effect	3	4.39	0.100	Uncorrelated^b^	N/A	N/A	N/A
						Random intercept	N/A	N/A	N/A
END LEAF DROP‐Intermediate	10	Year as fixed effect	6	0	0.980	Correlated^a^	year	121.9	0.38
		No fixed effect	5	7.83	0.020	Uncorrelated^b^	year	121.7	0
						Random intercept	year	113.7	37.28
END LEAF DROP‐Late	13	Year as fixed effect	6	0	1.000	Correlated^a^	year	144.2	0.22
		No fixed effect	5	15.57	0.000	Uncorrelated^b^	year	143.7	0
						Random intercept	year	134.3	44.12

*Note:* See Appendix C: Table C1a for further details.

^a^ Correlated random slope and intercept.

^b^ Unncorrelated random slope and intercept.

**TABLE C2a:** Estimated mean coefficients (and standard error) for intercepts and slopes in the top mixed‐effects model for temporal change in five spring phenological events for each seasonal group of species.

Event Seasonal group	Estimate	SE
BEGIN BUD SWELL Early Ohio buckeye sapling		
Intercept	82.6	1.0
Slope	−0.159	0.060
BEGIN BUD SWELL Late		
Intercept	103.5	1.2
Slope	−0.123	0.035
BEGIN BUD BURST Early Ohio buckeye sapling		
Intercept	90.5	0.5
Slope	0	N/A
BEGIN BUD BURST Late		
Intercept	114.7	1.1
Slope	−0.185	0.032
BEGIN LEAF EXPANSION Early O. buckeye sapling + tree		
Intercept	103.3	3.6
Slope	0	N/A
BEGIN LEAF EXPANSION Late		
Intercept	126.0	1.0
Slope	−0.202	0.037
END LEAF EXPANSION Early O. buckeye sapling + tree		
Intercept	115.6	2.2
Slope	0	N/A
END LEAF EXPANSION Intermediate		
Intercept	137.4	1.0
Slope	−0.216	0.044
END LEAF EXPANSION Late		
Intercept	156.0	3.8
Slope	−0.304	0.174
BEGIN FLOWERING Early		
Intercept	93.4	2.0
Slope	−0.208	0.053
BEGIN FLOWERING Late		
Intercept	116.7	1.9
Slope	−0.120	0.030

*Note:* The intercept can be interpreted as the relative phenology, where lesser values indicate earlier events or shorter durations. The slope is the coefficient representing change in days per year. For the variables with a slope of zero and no standard error (N/A), the null model which excluded year was found to be the top model.

**TABLE C2b:** Estimated mean coefficients (and standard error) for intercepts and slopes in the top mixed‐effects model for temporal change in four autumn events for each seasonal group of species.

Event Seasonal group	Estimate	SE
BEGIN LEAF COLORATION Early Ohio Buckeye sapling		
Intercept	195.8	2.3
Slope	0	N/A
BEGIN LEAF COLORATION Early Ohio Buckeye tree		
Intercept	246.4	2.2
Slope	−0.141	0.062
BEGIN LEAF COLORATION Intermediate		
Intercept	275.5	1.8
Slope	0.121	0.057
BEGIN LEAF COLORATION Late		
Intercept	288.2	1.1
Slope	0.334	0.049
END LEAF COLORATION Early Ohio Buckeye sapling		
Intercept	223.7	4.4
Slope	0	N/A
END LEAF COLORATION Early Ohio Buckeye tree		
Intercept	272.5	2.0
Slope	0	N/A
END LEAF COLORATION Intermediate		
Intercept	289.7	1.5
Slope	0.300	0.066
END LEAF COLORATION Late		
Intercept	303.2	1.2
Slope	0.322	0.034
BEGIN LEAF DROP Early Ohio Buckeye sapling		
Intercept	209.3	2.8
Slope	0	N/A
BEGIN LEAF DROP Early Ohio Buckeye tree		
Intercept	263.3	2.3
Slope	−0.508	0.066
BEGIN LEAF DROP Intermediate		
Intercept	289.9	1.6
Slope	0.137	0.061
BEGIN LEAF DROP Late		
Intercept	303.9	1.1
Slope	0.195	0.069
END LEAF DROP Early Ohio Buckeye sapling		
Intercept	227.9	4.8
Slope	0.243	0.131
END LEAF DROP Early Ohio Buckeye tree		
Intercept	282.4	2.1
Slope	−0.127	0.050
END LEAF DROP Intermediate		
Intercept	298.2	1.4
Slope	0.257	0.058
END LEAF DROP Late		
Intercept	312.9	1.2
Slope	0.284	0.048

*Note:* See Appendix C: Table C2a for details.

**TABLE C2c:** Estimated mean coefficients (and standard error) for intercepts and slopes in the top mixed‐effects model for temporal change in nine phase durations for each seasonal group of species.

Event Seasonal group	Estimate	SE
BUD SWELL DURATION Early Ohio Buckeye sapling		
Intercept	7.6	0.6
Slope	0.170	0.037
BUD SWELL DURATION Late		
Intercept	11.2	0.6
Slope	−0.060	0.021
BUD BURST DURATION Early Ohio Buckeye sapling		
Intercept	9.9	0.4
Slope	−0.046	0.027
BUD BURST DURATION Late		
Intercept	10.3	0.5
Slope	0	N/A
LEAF EXPAND DURATION Early O. buckeye sapling + tree		
Intercept	12.3	1.3
Slope	0	N/A
LEAF EXPAND DURATION Late		
Intercept	15.0	1.4
Slope	−0.031	0.017
FLOWERING DURATION Early		
Intercept	13.0	0.4
Slope	0	N/A
FLOWERING DURATION Late		
Intercept	12.7	0.6
Slope	−0.050	0.038
SPRING DURATION Early Ohio Buckeye sapling		
Intercept	30.8	1.0
Slope	0.155	0.063
SPRING DURATION Late		
Intercept	36.9	2.2
Slope	−0.120	0.046
LEAF COLORATION DURATION Early Ohio Buckeye sapling		
Intercept	25.1	2.6
Slope	0.210	0.088
LEAF COLORATION DURATION Early Ohio Buckeye tree		
Intercept	26.8	1.1
Slope	0.090	0.056
LEAF COLORATION DURATION Intermediate		
Intercept	13.7	0.9
Slope	0.171	0.065
LEAF COLORATION DURATION Late		
Intercept	14.3	0.8
Slope	0	N/A
LEAF DROP DURATION Early Ohio Buckeye sapling		
Intercept	22.0	1.9
Slope	0	N/A
LEAF DROP DURATION Early OB tree		
Intercept	19.4	1.2
Slope	0.379	0.062
LEAF DROP DURATION Intermediate		
Intercept	10.4	0.9
Slope	0.135	0.037
LEAF DROP DURATION Late		
Intercept	9.2	0.8
Slope	0.078	0.029
AUTUMN DURATION Early Ohio Buckeye sapling		
Intercept	31.2	2.6
Slope	0.315	0.095
AUTUMN DURATION Early Ohio Buckeye tree		
Intercept	36.4	0.9
Slope	0	N/A
AUTUMN DURATION Intermediate		
Intercept	21.2	0.9
Slope	0.131	0.065
AUTUMN DURATION Late		
Intercept	23.2	1.3
Slope	0	N/A
GROWING SEASON DURATION Early OB sapling + tree		
Intercept	117.0	20.6
Slope	0	N/A
GROWING SEASON DURATION Late		
Intercept	157.7	2.1
Slope	0.471	0.055

*Note:* See Appendix C: Table C2a for details.

**TABLE C3a:** Species‐level estimated coefficients for intercept and slope for each spring phenological event in the top mixed‐effects model.

Event	Begin Bud Swell		Begin Bud Burst		Begin Leaf Expansion		End Leaf Expansion		Begin Flowering	
Species	Intercept	Slope	Intercept	Slope	Intercept	Slope	Intercept	Slope	Intercept	Slope
*Tilia americana* Amer. basswood canopy tree	105.2	−0.080	115.8	−0.076	123.8	−0.043	134.4	−0.033	N/A	N/A
*Ulmus americana* American elm canopy tree	103.2	−0.100	114.9	−0.143	124.3	−0.138	134.9	−0.149	90.8	−0.208
*Carya cordiformis* Bitternut hickory canopy tree	104.9	−0.119	117.4	−0.187	129.5	−0.153	144.9	−0.274	127.4	−0.133
*Juglans nigra* Black walnut canopy tree	103.4	−0.076	113.5	−0.063	126.2	0.058	149.2	−0.020	126.4	−0.110
*Fraxinus quadrangulata* Blue ash canopy tree	107.2	−0.133	117.8	−0.159	127.5	−0.108	138.1	−0.151	108.7	−0.133
*Quercus macrocarpa* Bur oak canopy tree	105.5	−0.232	116.5	−0.230	126.2	−0.113	138.7	−0.076	119.8	−0.060
*Celtis occidentalis* Common hackberry canopy tree	99.4	0.070	112.9	−0.168	122.7	−0.178	135.1	−0.121	107.3	−0.197
*Fraxinuus pennsylvanica* Green ash canopy tree	108.8	−0.171	119.3	−0.272	129.6	−0.301	141.4	−0.380	116.4	−0.146
*Gymnocladus dioicus* Kentucky coffeetree canopy tree	108.6	−0.137	118.8	−0.112	131.5	−0.080	150.1	−0.115	N/A	N/A
*Quercus rubra* Northern red oak canopy tree	105.3	−0.262	115.7	−0.262	124.2	−0.125	136.5	−0.041	117.6	−0.099
*Aesculus glabra* Ohio buckeye canopy tree	92.2	−0.061	100.3	−0.046	106.8	0	117.8	0	116.8	−0.115
*Carya laciniosa* Shellbark hickory canopy tree	104.4	0.013	117.0	−0.175	125.9	−0.140	138.3	−0.164	124.9	−0.140
*Ulmus rubra* Slippery elm canopy tree	109.9	−0.193	120.6	−0.232	129.5	−0.186	140.0	−0.163	92.3	−0.208
*Acer saccharum* Sugar maple canopy tree	101.5	−0.055	113.4	−0.067	121.2	−0.053	130.8	−0.086	108.4	−0.025
*Fraxinus americana* White ash canopy tree	109.9	−0.220	120.0	−0.312	128.7	−0.338	139.6	−0.353	115.3	−0.154
*Carpinus caroliniana* Amer. hornbeam treelet	94.3	0.028	107.4	−0.048	119.3	−0.070	135.8	−0.130	108.3	−0.037
*Asimina triloba* Pawpaw treelet	98.4	0.052	119.1	−0.278	137.5	−0.364	162.8	−0.379	123.3	−0.113
*Zanthoxylum americanum* Common pricklyash shrub	97.5	−0.203	109.4	−0.304	123.8	−0.485	162.1	−0.740	113.2	−0.217
*Lindera benzoin* Northern spicebush shrub	90.3	0.006	105.6	−0.045	122.8	−0.428	141.2	−0.500	97.0	−0.208
*Toxicodendron radicans* Eastern poison ivy vine	105.6	−0.195	114.5	−0.253	125.2	−0.305	142.1	−0.461	N/A	N/A
*Vitis* spp. Grape species mixture. vine	114.5	−0.532	123.6	−0.514	132.4	−0.409	N/A	N/A	N/A	N/A
*Parthenocissus quinquefolia* Virginia creeper vine	109.1	−0.333	118.6	−0.399	126.1	−0.391	137.9	−0.442	N/A	N/A
*Fraxinus quadrangulata* Blue ash sapling	100.5	0.098	110.2	−0.021	122.2	−0.196	134.5	−0.273	N/A	N/A
*Aesculus glabra* Ohio buckeye sapling	82.6	−0.159	90.5	0	99.8	0	113.5	0	N/A	N/A
*Acer saccharum* Sugar maple sapling	103.5	−0.123	110.3	−0.086	118.3	−0.101	129.1	−0.094	N/A	N/A

*Note:* The intercept can be interpreted as the relative phenology, with lesser values indicating earlier events or shorter durations of phases. The slope is the coefficient representing change in days per year. The coefficients are derived from models calculated using raw (not standardized) weather variables. Slope values of zero represent cases where the null model which excluded year was found to be the top model. Abbreviations for species used in Figures 2, 3, and 5 are in Appendix A: Table A6.

**TABLE C3b:** Species‐level estimated coefficients for intercept and slope for each autumn phenological event in the top mixed‐effects model.

Event	Begin Leaf Coloration		End Leaf Coloration		Begin Leaf Drop		End Leaf Drop	
Species	Intercept	Slope	Intercept	Slope	Intercept	Slope	Intercept	Slope
*Tilia americana* Amer. basswood canopy tree	281.3	−0.144	296.3	0.297	289.5	0.012	301.2	0.258
*Ulmus americana* American elm canopy tree	291.1	0.458	306.7	0.359	305.7	0.325	315.6	0.378
*Carya cordiformis* Bitternut hickory canopy tree	286.3	0.481	298.8	0.481	299.9	0.566	311.4	0.580
*Juglans nigra* Black walnut canopy tree	270.0	0.244	288.0	0. 441	281.5	0.143	295.5	0.365
*Fraxinus quadrangulata* Blue ash canopy tree	290.9	0.397	303.4	0.297	303.3	0.128	310.8	0.222
*Quercus macrocarpa* Bur oak canopy tree	290.7	0.264	306.2	0.261	303.5	0.149	314.0	0.181
*Celtis occidentalis* Common hackberry canopy tree	287.4	−0.040	306.0	0.272	304.6	−0.148	316.0	0.123
*Fraxinuus pennsylvanica* Green ash canopy tree	279.1	0.148	290.0	0.200	288.9	0.081	296.9	0.149
*Gymnocladus dioicus* Kentucky coffeetree canopy tree	276.2	0.151	288.2	0.418	286.6	−0.025	295.6	0.347
*Quercus rubra* Northern red oak canopy tree	289.3	0.565	308.2	0.422	310.2	0.370	322.6	0.318
*Aesculus glabra* Ohio buckeye canopy tree	246.4	−0.141	272.5	0	263.3	−0.508	282.4	−0.127
*Carya laciniosa* Shellbark hickory canopy tree	280.8	0.403	291.9	0.464	295.2	0.396	309.1	0.454
*Ulmus rubra* Slippery elm canopy tree	288.2	0.267	306.3	0.251	296.9	0.154	313.2	0.214
*Acer saccharum* Sugar maple canopy tree	286.8	0.152	299.3	0.246	300.7	0.105	309.1	0.184
*Fraxinus americana* White ash canopy tree	276.0	0.042	288.5	0.051	288.9	−0.140	296.5	0.007
*Carpinus caroliniana* Amer. hornbeam treelet	283.4	0.540	296.4	0.482	295.0	0.405	304.3	0.414
*Asimina triloba* Pawpaw treelet	289.2	0.449	302.0	0.402	302.3	0.310	309.3	0.343
*Zanthoxylum americanum* Common pricklyash shrub	287.3	0.249	304.2	0.257	302.0	−0.063	312.4	0.112
*Lindera benzoin* Northern spicebush shrub	284.2	0.195	297.5	0.170	296.4	0.019	303.8	0.141
*Toxicodendron radicans* Eastern poison ivy vine	276.3	0.120	289.5	0.148	288.5	0.091	298.4	0.097
*Vitis* spp. grape species mixture vine	277.3	0.222	292.2	0.530	288.2	0.422	298.9	0.493
*Parthenocissus quinquefolia* Virginia creeper vine	267.6	0.182	281.2	0.275	283.8	0.084	290.9	0.300
*Fraxinus quadrangulata* Blue ash sapling	295.2	0.352	307.6	0.296	306.3	0.238	313.1	0.325
*Aesculus glabra* Ohio buckeye sapling	195.8	0	223.7	0	209.3	0	227.9	0.243
*Acer saccharum* Sugar maple sapling	291.5	0.276	303.1	0.180	304.9	0.167	310.6	0.265

*Note:* See Appendix C: Table C3a for details.

**TABLE C3c:** Species‐level estimated coefficients for intercept and slope for each spring phenological stage duration in the top mixed‐effects model.

Phase	Bud swell duration		Bud burst duration		Leaf expansion duration		Flowering duration		Spring phenology duration	
Species	Intercept	Slope	Intercept	Slope	Intercept	Slope	Intercept	Slope	Intercept	Slope
*Tilia americana* Amer. basswood canopy tree	10.5	0.005	7.9	0.032	10.7	0.003	N/A	N/A	29.1	0.046
*Ulmus americana* American elm canopy tree	11.8	−0.031	9.1	0.004	10.6	−0.017	13.5	0	31.6	−0.041
*Carya cordiformis* Bitternut hickory canopy tree	12.4	−0.062	12.4	0.004	15.4	−0.068	13.2	−0.062	40.8	−0.167
*Juglans nigra* Black walnut canopy tree	10.1	0.013	12.6	0.120	23.1	−0.048	13.1	0.015	45.8	0.087
*Fraxinus quadrangulata* Blue ash canopy tree	10.9	−0.025	9.6	0.012	10.9	−0.016	13.1	0.139	31.4	−0.033
*Quercus macrocarpa* Bur oak canopy tree	11.0	−0.001	9.6	0.123	12.8	0.025	13.8	−0.178	33.2	0.160
*Celtis occidentalis* Common hackberry canopy tree	13.5	−0.236	9.8	−0.003	12.7	0.036	9.7	−0.108	35.8	−0.195
*Fraxinuus pennsylvanica* Green ash canopy tree	10.6	−0.101	10.8	−0.060	11.2	−0.030	12.2	−0.111	33.0	−0.232
*Gymnocladus dioicus* Kentucky coffeetree canopy tree	10.2	0.026	12.9	0.012	18.3	−0.034	N/A	N/A	41.4	0.010
*Quercus rubra* Northern red oak canopy tree	10.4	−0.005	8.3	0.149	12.7	0.057	14.7	−0.164	31.1	0.231
*Aesculus glabra* Ohio buckeye canopy tree	8.0	0.018	7.3	0.010	11.0	0	17.1	−0.101	26.8	−0.016
*Carya laciniosa* Shellbark hickory canopy tree	12.5	−0.188	8.9	0.035	12.4	−0.023	13.9	−0.029	34.0	−0.184
*Ulmus rubra* Slippery elm canopy tree	10.8	−0.035	9.0	0.032	10.6	0.019	12.6	0	30.3	0.030
*Acer saccharum* Sugar maple canopy tree	11.8	−0.009	7.6	0.018	9.6	−0.032	13.3	−0.150	29.1	−0.029
*Fraxinus americana* White ash canopy tree	10.0	−0.084	8.8	−0.028	10.8	−0.010	11.0	−0.139	29.7	−0.138
*Carpinus caroliniana* Amer. hornbeam treelet	13.0	−0.072	11.8	−0.011	16.6	−0.064	10.9	−0.083	41.5	−0.159
*Asimina triloba* Pawpaw treelet	20.7	−0.332	18.9	−0.132	25.1	−0.016	12.2	0.039	64.5	−0.443
*Zanthoxylum americanum* Common pricklyash shrub	11.7	−0.077	14.2	−0.147	37.6	−0.178	10.2	0.240	64.0	−0.457
*Lindera benzoin* Northern spicebush shrub	15.4	−0.065	17.3	−0.388	18.4	−0.070	12.7	0	51.0	−0.492
*Toxicodendron radicans* Eastern poison ivy vine	8.8	−0.059	10.7	−0.037	16.4	−0.105	N/A	N/A	36.6	−0.270
*Vitis* spp. grape species mixture vine	9.2	−0.003	8.8	0.125	N/A	N/A	N/A	N/A	N/A	N/A
*Parthenocissus quinquefolia* Virginia creeper vine	9.5	−0.075	7.4	0.032	11.7	−0.041	N/A	N/A	28.7	−0.112
*Fraxinus quadrangulata* Blue ash sapling	**9.6**	−0.086	12.1	−0.200	12.2	−0.064	N/A	N/A	34.1	−0.398
*Aesculus glabra* Ohio buckeye sapling	7.6	0.170	9.9	−0.046	13.6	0	N/A	N/A	30.8	0.155
*Acer saccharum* Sugar maple sapling	6.8	0.041	7.6	0.002	11.0	−0.010	N/A	N/A	25.3	0.034

*Note:* See Appendix C: Table C3a for details.

**TABLE C3d:** Species‐level estimated coefficients for intercept and slope for each autumn phenological phase and total growing season duration in the top mixed‐effects model.

Phase	Leaf coloration duration		Leaf drop duration		Autumn phenology duration		Growing season duration	
Species	Intercept	Slope	Intercept	Slope	Intercept	Slope	Intercept	Slope
*Tilia americana* Amer. basswood canopy tree	14.0	0.459	11.9	0.241	19.8	0.410	158.0	−0.114
*Ulmus americana* American elm canopy tree	15.2	−0.030	9.7	0.078	24.2	−0.023	167.4	0.583
*Carya cordiformis* Bitternut hickory canopy tree	12.3	0.027	11.1	0.068	24.4	0.127	156.1	0.646
*Juglans nigra* Black walnut canopy tree	17.9	0.197	14.0	0.222	25.3	0.126	143.7	0.191
*Fraxinus quadrangulata* Blue ash canopy tree	11.2	−0.093	7.4	0.058	17.0	−0.099	164.6	0.466
*Quercus macrocarpa* Bur oak canopy tree	15.5	0.001	10.5	0.028	23.1	−0.077	164.6	0.369
*Celtis occidentalis* Common hackberry canopy tree	18.7	0.304	11.9	0.244	28.9	0.147	164.6	0.135
*Fraxinuus pennsylvanica* Green ash canopy tree	11.3	0.009	8.4	0.067	18.1	−0.038	149.9	0.528
*Gymnocladus dioicus* Kentucky coffeetree canopy tree	12.1	0.261	9.4	0.353	19.4	0.192	144.8	0.233
*Quercus rubra* Northern red oak canopy tree	18.8	−0.115	12.3	−0.030	33.4	−0.223	165.3	0.678
*Aesculus glabra* Ohio buckeye canopy tree	26.8	0.090	19.4	0.379	36.4	0	137.4	0
*Carya laciniosa* Shellbark hickory canopy tree	12.0	0.041	12.8	0.086	28.1	0.057	154.4	0.549
*Ulmus rubra* Slippery elm canopy tree	18.1	−0.031	15.6	0.083	24.9	−0.046	158.8	0.455
*Acer saccharum* Sugar maple canopy tree	12.5	0.076	8.6	0.065	22.1	0.031	165.6	0.197
*Fraxinus americana* White ash canopy tree	12.5	0.019	7.9	0.137	20.5	−0.026	147.0	0.446
*Carpinus caroliniana* Amer. hornbeam treelet	12.9	−0.055	9.4	0.012	21.6	−0.128	163.9	0.601
*Asimina triloba* Pawpaw treelet	12.7	−0.037	6.7	0.036	19.5	−0.078	151.5	0.818
*Zanthoxylum americanum* Common pricklyash shrub	17.0	0.017	10.9	0.131	25.0	−0.133	163.7	0.643
*Lindera benzoin* Northern spicebush shrub	13.8	−0.027	7.5	0.114	20.1	−0.054	161.1	0.625
*Toxicodendron radicans* Eastern poison ivy vine	13.2	0.039	10.0	0.021	21.9	−0.007	150.6	0.524
*Vitis* spp. Grape species mixture vine	14.9	0.305	10.8	0.086	21.6	0.267	144.3	0.630
*Parthenocissus quinquefolia* Virginia creeper vine	13.7	0.081	7.3	0.199	23.0	0.125	140.4	0.696
*Fraxinus quadrangulata* Blue ash sapling	12.2	−0.039	6.8	0.087	17.6	−0.004	173.2	0.578
*Aesculus glabra* Ohio buckeye sapling	25.1	0.210	22.0	0	31.2	0.315	96.5	0
*Acer saccharum* Sugar maple sapling	11.4	−0.106	5.6	0.092	18.6	−0.001	173.7	0.361

*Note:* See Appendix C: Table C3a for details.

**TABLE C4a:** Model selection for temporal change in duration of each of five spring phenophases for seasonal groups of species (and growth forms) in Trelease Woods (1993–2021).

Phase—Seasonal group	*N*	Fixed Effects				Random Effects			
		Model	df	ΔAICc	Akaike weight	Model	Fixed effect	df	ΔcAIC
BUD SWELL DURATION‐Early Ohio buckeye sapling	1	Year as fixed effect	4	0	1.000	Correlated^a^	N/A	N/A	N/A
		No fixed effect	3	18.26	0.000	Uncorrelated^b^	N/A	N/A	N/A
						Random intercept	N/A	N/A	N/A
BUD SWELL DURATION‐Late	24	Year as fixed effect	6	0	0.939	Correlated^a^	Year	43.6	0
		No fixed effect	5	5.46	0.061	Uncorrelated^b^	Year	43.8	0.68
						Random intercept	Year	27.4	163.17
BUD BURST DURATION‐Early Ohio buckeye sapling	1	Year as fixed effect	4	0	0.605	Correlated^a^	N/A	N/A	N/A
		No fixed effect	3	0.85	0.395	Uncorrelated^b^	N/A	N/A	N/A
						Random intercept	N/A	N/A	N/A
BUD BURST DURATION‐Late	24	Year as fixed effect	6	1.69	0.300	Correlated^a^	(None)	66.4	0
		No fixed effect	5	0	0.700	Uncorrelated^b^	(None)	66.8	0.95
						Random intercept	(None)	53.9	247.64
LEAF EXPAND DURATION‐Early Ohio buckeye sapling + tree	2	Year as fixed effect	5	1.74	0.295	Correlated^a^	N/A	N/A	N/A
		No fixed effect	4	0	0.705	Uncorrelated^b^	N/A	N/A	N/A
						Random intercept	N/A	N/A	N/A
LEAF EXPAND DURATION‐Late	23	Year as fixed effect	6	0	0.762	Correlated^a^	Year	152.6	0
		No fixed effect	5	2.32	0.238	Uncorrelated^b^	Year	151.3	2.42
						Random intercept	Year	139.4	25.84
FLOWERING DURATION‐Early	3	Year as fixed effect	5	1.95	0.273	Correlated^a^	N/A	N/A	N/A
		No fixed effect	4	0	0.727	Uncorrelated^b^	N/A	N/A	N/A
						Random intercept	N/A	N/A	N/A
FLOWERING DURATION‐Late	14	Year as fixed effect	6	0	0.505	Correlated^a^	Year	78.4	0
		No fixed effect	5	0.04	0.495	Uncorrelated^b^	Year	77.5	0.17
						Random intercept	Year	74.9	64.24
SPRING DURATION‐Early. Ohio buckeye sapling	1	Year as fixed effect	4	0	0.884	Correlated^a^	N/A	N/A	N/A
		No fixed effect	3	4.06	0.116	Uncorrelated^b^	N/A	N/A	N/A
						Random intercept	N/A	N/A	N/A
SPRING DURATION‐Late	24	Year as fixed effect	6	0	0.913	Correlated^a^	Year	137.4	0
		No fixed effect	5	4.69	0.087	Uncorrelated^b^	Year	136.4	1.57
						Random intercept	Year	110.5	208.79

*Note:* Model selection was conducted in two steps. Step 1 evaluated year as a fixed effect; year was used as a predictor if its model AICc value was less than that of the null model. Step 2 evaluated the structure of random effects, using the fixed effects structure from Step 1. Step 2 was not done when there were fewer than four species because random slopes could not be tested, and the model with random intercept was immediately chosen after Step 1. The model with the lowest conditional AIC (cAIC) was considered the top model. *N* represents the number of species (counting saplings and canopy trees separately for three species) in each analysis.

^a^ Correlated random slope and intercept.

^b^ Uncorrelated random slope and intercept.

**TABLE C4b:** Model selection for temporal change in duration of each of four autumn phenophases for seasonal groups of species (and growth forms) in Trelease Woods (1993–2021).

Phase—Seasonal group	*N*	Fixed Effects				Random Effects			
		Model	df	ΔAICc	Akaike weight	Model	Fixed effect	df	ΔcAIC
LEAF COLORATION DURATION‐ Early Ohio buckeye sapling	1	Year as fixed effect	4	0	0.857	Correlated^a^	N/A	N/A	N/A
		No fixed effect	3	3.58	0.143	Uncorrelated^b^	N/A	N/A	N/A
						Random intercept	N/A	N/A	N/A
LEAF COLORATION DURATION‐ Early Ohio buckeye tree	1	Year as fixed effect	4	0	0.559	Correlated^a^	N/A	N/A	N/A
		No fixed effect	3	0.47	0.441	Uncorrelated^b^	N/A	N/A	N/A
						Random intercept	N/A	N/A	N/A
LEAF COLORATION DURATION‐ Intermediate	8	Year as fixed effect	6	0	0.832	Correlated^a^	year	70.4	0.07
		No fixed effect	5	3.19	0.168	Uncorrelated^b^	year	70.7	0
						Random intercept	year	64.0	61.87
LEAF COLORATION DURATION‐ Late	15	Year as fixed effect	6	1.97	0.272	Correlated^a^	(none)	127.7	0.11
		No fixed effect	5	0	0.728	Uncorrelated^b^	(none)	128.1	0.
						Random intercept	(none)	114.5	103.80
LEAF DROP DURATION‐ Early Ohio buckeye sapling	1	Year as fixed effect	4	0.84	0.396	Correlated^a^	N/A	N/A	N/A
		No fixed effect	3	0	0.604	Uncorrelated^b^	N/A	N/A	N/A
						Random intercept	N/A	N/A	N/A
LEAF DROP DURATION‐ Early Ohio buckeye tree	1	Year as fixed effect	4	0	1.000	Correlated^a^	N/A	N/A	N/A
		No fixed effect	3	33.55	0.000	Uncorrelated^b^	N/A	N/A	N/A
						Random intercept	N/A	N/A	N/A
LEAF DROP DURATION‐ Intermediate	12	Year as fixed effect	6	0	0.974	Correlated^a^	year	130.3	0.49
		No fixed effect	5	7.23	0.026	Uncorrelated^b^	year	129.9	0
						Random intercept	year	121.2	51.32
LEAF DROP DURATION‐ Late	11	Year as fixed effect	6	0	0.882	Correlated^a^	year	81.6	0.58
		No fixed effect	5	4.02	0.118	Uncorrelated^b^	year	81.3	0
						Random intercept	year	73.6	42.13
AUTUMN DURATION‐ Early Ohio buckeye sapling	1	Year as fixed effect	4	0	0.988	Correlated^a^	N/A	N/A	N/A
		No fixed effect	3	8.79	0.012	Uncorrelated^b^	N/A	N/A	N/A
						Random intercept	N/A	N/A	N/A
AUTUMN DURATION‐ Early Ohio buckeye tree	1	Year as fixed effect	4	2.00	0.269	Correlated^a^	N/A	N/A	N/A
		No fixed effect	3	0	0.731	Uncorrelated^b^	N/A	N/A	N/A
						Random intercept	N/A	N/A	N/A
AUTUMN DURATION‐ Intermediate	8	Year as fixed effect	6	0	0.678	Correlated^a^	(none)	76.0	0.39
		No fixed effect	5	1.49	0.322	Uncorrelated^b^	(none)	75.7	0
						Random intercept	(none)	68.9	53.43
AUTUMN DURATION‐ Late	15	Year as fixed effect	6	0.25	0.469	Correlated^a^	(none)	157.8	0.58
		No fixed effect	5	0	0.531	Uncorrelated^b^	(none)	57.5	0
						Random intercept	(none)	146.3	49.36
GROWING SEASON DURATION‐ Early O. buckeye sapling + tree	2	Year as fixed effect	5	0.96	0.382	Correlated^a^	N/A	N/A	N/A
		No fixed effect	4	0	0.618	Uncorrelated^b^	N/A	N/A	N/A
						Random intercept	N/A	N/A	N/A
GROWING SEASON DURATION‐ Late	24	Year as fixed effect	6	0	1.000	Correlated^a^	year	246.9	0.28
		No fixed effect	5	33.50	0.000	Uncorrelated^b^	year	246.7	0
						Random intercept	year	225.7	215.66

*Note:* See Appendix C: Table C4a for details.

^a^ Correlated random slope and intercept.

^b^ Uncorrelated random slope and intercept.

**TABLE C5a:** The top five mixed‐effects models with weather predicting each of five spring phenological events in a combined 11 seasonal groups.

Event Seasonal group	Fixed Effects in Model	AICc	ΔAICc
BEGIN BUD SWELL Early Ohio buckeye sapling	GDD2.m1 + CDD4.m3 + Precip.PrevSummer	1764.93	0
	GDD2.m1 + CDD4.m3 + CDD8.s1	1792.61	27.68
	GDD2.m1 + CDD4.m3 + GDD10.m2	1796.45	31.52
	GDD2.m1 + CDD4.m3 + Mean T.PrevSummer	1797.04	32.12
	GDD2.m1 + CDD4.m3 + Precip.S1	1806.33	41.40
BEGIN BUD SWELL Late species	GDD2.m1 + CDD4.s2 + CDD8.s1	43,672.73	0
	GDD2.m1 + CDD8.s1 + Flood.m3	43,802.42	129.69
	GDD2.m1 + CDD4.s2 + CDD6.m6	43,839.57	166.84
	GDD2.m1 + CDD4.s2 + Precip.m4	43,846.49	173.76
	GDD2.m1 + CDD8.s1 + CDD6.m6	43,853.53	180.8
BEGIN BUD BURST Early Ohio Buckeye sapling	GDD0.m1 + Begin Bud Swell + Rain Days.s1	1587.78	0
	GDD0.m1 + Begin Bud Swell + GDD10.m2	1598.77	10.99
	GDD0.m1 + Begin Bud Swell + Precip.m2	1602.57	14.79
	GDD0.m1 + Begin Bud Swell + MeanT.PrevAutumn	1606.90	19.12
	GDD0.m1 + Begin Bud Swell + Flood.m3	1607.41	19.63
BEGIN BUD BURST Late species	Begin Bud Swell + GDD4.m1 + Precip.PrevAutumn	39,742.26	0
	Begin Bud Swell + GDD4.m1 + Rain Days.m3	39,983.79	241.53
	Begin Bud Swell + GDD4.m1 + Flood.m1	40,239.62	497.36
	Begin Bud Swell + GDD4.m1 + GDD0.m3	40,281.26	539.00
	Begin Bud Swell + GDD4.m1 + GDD12.m2	40299.11	556.85
BEGIN LEAF EXPANSION Early O. buckeye sapling + tree	Begin Bud Burst + GDD10.s1 + Flood.m1	3588.78	0
	Begin Bud Burst + GDD10.s1 + Flood.m4	3590.59	1.81
	Begin Bud Burst + GDD10.s1 + Flood.m2	3590.96	2.18
	Begin Bud Burst + GDD10.s1 + GDD12.m2	3592.06	3.28
	Begin Bud Burst + GDD10.s1 + MeanT.PrevSummer	3594.10	5.32
BEGIN LEAF EXPANSION Late species	Begin Bud Burst + GDD8.s1 + Precip.PrevAutumn	36,380.46	0
	Begin Bud Burst + GDD8.s1 + Flood.m4	36,710.14	329.68
	Begin Bud Burst + GDD8.s1 + Precip.PrevSummer	36,826.87	446.41
	Begin Bud Burst + GDD8.s1 + Rain Days.m2	36,867.86	487.40
	Begin Bud Burst + GDD8.s1 + Flood.s1	36,899.44	518.98
END LEAF EXPANSION Early O Buckeye sapling + tree	GDD2.s1 + Rain Days.m3 + Begin Leaf Expansion	4454.91	0
	GDD2.s1 + Rain Days.m3 + Begin Leaf Burst	4469.61	14.7
	GDD2.s1 + Rain Days.m3 + Flood.m1	4472.08	17.17
	GDD2.s1 + Rain Days.m3 + Mean T.s2	4483.84	28.93
	GDD2.s1 + Rain Days.m3 + Begin Bud Swell	4489.37	34.46
END LEAF EXPANSION Intermediate species	Begin Leaf Expansion + GDD6.s1 + Flood.m3	29,083.75	0
	Begin Leaf Expansion + GDD6.s1 + Precip.s2	29,156.87	73.12
	Begin Leaf Expansion + GDD6.s1 + Rain.m2	29,166.39	82.64
	Begin Leaf Expansion + GDD6.s1 + Mean T.s2	29,166.62	82.87
	Begin Leaf Expansion + GDD6.s1 + GDD12.m3	29,214.73	130.98
END LEAF EXPANSION Late species	Rain Days.s1 + Begin Leaf Expansion + GDD2.s1	10,054.76	0
	GDD12.m3 + Begin Leaf Expansion + GDD2.s1	10,103.21	48.45
	Begin Leaf Expansion + GDD2.s1 + Flood.m1	10,144.21	89.45
	Begin Leaf Expansion + GDD2.s1 + Flood.m2	10,153.23	98.47
	Begin Leaf Expansion + GDD2.s1+ Flood.s2	10,154.03	99.27
BEGIN FLOWERING Early species	CCD0.s1 + GDD12.m3 + GDD10.m5	3253.39	0
	GDD0.s1 + GDD12.m3 + Rain Days.m3	3258.57	5.18
	GDD0.s1 + GDD10.m5 + Rain Days.s1	3259.87	6.48
	GDD0.s1 + GDD10.m5 + Mean T.m3	3260.34	6.95
	GDD0.s1 + GDD12.m3 + Mean T.PrevAutumn	3263.70	10.31
BEGIN FLOWERING Late species	Begin Bud Burst + GDD8.s1 + CDD8.m2	18,129.57	0
	GDD8.s1 + Begin Bud Swell + Rain Days.m3	18,152.18	22.61
	Begin Bud Swell + GDD8.s1 + Flood.m3	18,260.09	130.52
	Begin Bud Burst + GDD8.s1 + Flood.m1	18,263.66	134.09
	Begin Bud Swell + GDD8.s1 + CDD8.m2	18,269.29	139.72

*Note:* Models were evaluated using AICc, and the top model (lowest AICc) is listed first. Within each model, fixed effects are in order of decreasing *t*‐value. See Appendix C: Table C6 for details of the top model. For all models, standardized values of fixed effects were used (transformed to have a mean of zero and standard deviation of one because of the great difference in scales and units). See Appendix A: Table A5 for species in each seasonal group. See Appendix A Tables A6, A7a,andA7b, for weather variable names and descriptions and Stop Dates (after which no weather values are used). The label “m” refers to “month” (30‐day period at some interval before Stop Date) and “s” to “season” (60‐day period at some interval before Stop Date). For example, m3 = month 3 = 60–90 days before Stop Date and s2 = season 2 = 60–120 days before Stop Date. Stop date is the mid‐point of the range of mean dates of the event among the species in a seasonal group. The abbreviations “PrevSummer” and “PrevAutumn” refer to weather variables from the previous year's summer or autumn.

**TABLE C5b:** The top five mixed‐effects models with weather predicting each of two autumn phenological events. The two events were evaluated in a combined eight seasonal groups.

Event Seasonal group	Fixed effects in model	AICc	ΔAICc
BEGIN LEAF COLORATION Early Ohio Buckeye Sapling	Rain Days.m4 + Mean T.s2 + Hot Days.s1	2191.41	0
	Rain Days.m4 + Mean T.s2 + Mean T.m2	2197.55	6.14
	Rain Days.m4 + Mean T.s2 + Rain Days.s1	2199.11	7.7
	Rain Days.m4 + Hot Days.s1 + Mean T.m2	2199.81	8.4
	Rain Days.m4 + Mean T.s2 + Precip.s2	2202.05	10.64
BEGIN LEAF COLORATION Early Ohio Buckeye Tree	Mean T.s1 + Drought.m4 + Rain Days.s1	3223.21	0
	Drought.m4 + Mean T.s1 + Precip.m2	3228.30	5.09
	Drought.m4 + Precip.m2 + Mean T.s3	3228.68	5.47
	Mean T.s1 + Drought.m4 + Mean T.m4	3230.53	7.32
	Drought.m4 + Mean T.s1 + Mean T.s3	3231.52	8.31
BEGIN LEAF COLORATION Intermediate Species	Min T.m1 + Drought.m3 + Precip.s2	16,706.89	0
	Min T.m1 + HDD8.m1 + Precip.s2	16,733.65	26.76
	Min T.m1 + HDD8.m1 + Min T.s3	16,751.73	44.84
	Min T.m1 + Hot Days.s1 + Min T.m3	16,752.19	45.3
	Min T.m1 + Min T.s3 + Precip.s2	16,752.52	45.63
BEGIN LEAF COLORATION Late Species	Min T.s1 + Drought.m3 + Min T.m6	28,262.09	0
	Min T.s1 + Min T.m6 + Mean T.m5	28,354.09	92
	Min T.s1 + Min T.m6 + Drought.m1	28,362.54	100.45
	Min T.s1 + Min T.m6 + HDD_i8_.m2	28,380.22	118.13
	Min T.s1 + Min T.m6 + Hot Days.s2	28,406.95	144.86
BEGIN LEAF DROP Early Ohio Buckeye Sapling	Begin Leaf Color + Hot Days.m1 + Mean T.s3	1989.59	67.05
	Begin Leaf Color + Hot Days.m1 + Hot Days.m2	1991.08	68.54
	Begin Leaf Color + Hot Days.m1 + End Leaf Expand	1992.00	69.46
	Begin Leaf Color + Hot Days.m1 + Drought.m4	1922.54	0
	Begin Leaf Color + Drought.s1 + Drought.m4	1992.70	70.16
BEGIN LEAF DROP Early Ohio Buckeye tree	Begin Leaf Color + Mean T.m4 + Rain Days.m2	2804.42	0
	Begin Leaf Color + Mean T.m4 + Mean T.m5	2810.35	5.93
	Begin Leaf Color + Mean T.m4 + Rain Days.m4	2821.81	17.39
	Begin Leaf Color + Mean T.m4 + End Leaf Expand	2824.07	19.65
	Begin Leaf Color + Mean T.m4 + Mean T.m6	2824.29	19.87
BEGIN LEAF DROP Intermediate Species	Begin Leaf Color + Min T.s1 + Min T.m6	22,866.17	0
	Begin Leaf Color + Min T.s1 + Precip.m3	22,899.72	33.55
	Begin Leaf Color + Min T.s1 + Hot Days.m3	22,913.25	47.08
	Begin Leaf Color + Min T.s1 + Hot Days.m2	22,920.81	54.64
	Begin Leaf Color + Min T.s1 + Precip.m1	22,928.29	62.12
BEGIN LEAF DROP Late Species	Begin Leaf Color + Min T.m6 + Mean T.m2	18,276.13	0
	Begin Leaf Color + Min T.m6 + Precip.m4	18,277.43	1.3
	Begin Leaf Color + Min T.m6 + Hot Days.m1	18,277.91	1.78
	Begin Leaf Color +End Leaf Expand +Hot Days.m1	18,280.37	4.24
	Begin Leaf Color + End Leaf Expand + Precip.m4	18,287.45	11.32

*Note:* See Appendix A: Tables A6, A7a, and A7b for weather variable names and descriptions and Stop Dates. See Appendix C: Table C5a for other details.

**TABLE C6:** Coefficients for three fixed effects included in the top linear mixed‐effects models predicting event date as a function of weather or legacy events. Analyses were completed for each of seven phenological events, each separated into multiple seasonal groups (19 total analyses).

Event Seasonal group	Predictor	Raw coefficient	Model Coefficient	SE	*t*‐value	Occurrence
BEGIN BUD SWELL Early OB sapling	(Intercept)	91.46	80.42	0.29		
	GDD2.m1	−0.12	−5.70	0.31	−18.29	top +4
	CDD4.m3	0.03	2.82	0.31	8.98	top +4
	Precip.PrevSummer	−0.02	−2.07	0.30	−6.90	top +0
BEGIN BUD SWELL Late	(Intercept)	107.35	102.05	1.05		
	GDD2.m1	−0.08	−4.89	0.24	−20.26	top +4
	CDD4.s2	0.01	1.21	0.12	10.46	top +2
	CDD8.s1	0.01	0.95	0.20	4.85	top +0
BEGIN BUD BURST Early OB sapling	(Intercept)	72.02	90.48	0.43		
	GDD0.m1	−0.07	−4.66	0.31	−15.16	top +4
	Begin Bud Burst	0.31	2.71	0.31	8.67	top +4
	Rain Days.s1	0.43	1.19	0.22	5.52	top +0
BEGIN BUD BURST Late	(Intercept)	75.40	112.14	0.50		
	Begin Bud Swell	0.51	4.85	0.24	20.58	top +4
	GDD4.m1	−0.07	−3.30	0.21	−16.07	top +4
	Precip.PrevAutumn	−0.02	−1.09	0.13	−8.67	top +0
BEGIN LEAF EXPAND Early OB sapling + tree	(Intercept)	47.08	103.87	0.70		
	Begin Bud Burst	0.64	5.71	0.18	30.88	top +4
	GDD10.s1	−0.08	−2.70	0.16	−16.43	top +4
	Flood.m1	−0.41	−0.36	0.11	−3.34	top +0
BEGIN LEAF EXPAND Late	(Intercept)	59.44	123.50	0.55	223.82	
	Begin Bud Burst	0.66	6.01	0.16	36.98	top +4
	GDD8.S1	−0.03	−2.04	0.15	−13.89	top +4
	Precip.PrevAutumn	−0.01	−0.98	0.12	−8.16	top +0
END LEAF EXPAND Early OB sap + tree	(Intercept)	110.76	115.84	1.22		
	GDD2.s1	−0.06	−4.7	0.33	−14.48	top +4
	Rain.m3	0.82	−1.77	0.20	−8.97	top +4
	Begin Leaf Expand	0.29	2.49	0.36	−6.94	top +0
END LEAF EXPAND Intermediate	(Intercept)	54.69	134.55	0.53		
	Begin Leaf Expand	0.71	6.20	0.18	34.15	top +4
	GDD6.s1	−0.02	−1.65	0.18	−9.04	top +4
	Flood.m3	0.58	0.44	0.25	1.77	top +0
END LEAF EXPAND Late	(Intercept)	106.77	153.25	2.98		
	Rain.s1	0.50	1.89	0.18	10.26	top +0
	Begin Leaf Expand	−0.51	4.97	0.83	5.97	top +4
	GDD2.s1	−0.04	−2.71	0.68	−3.97	top +3
BEGIN FLOWERING Early	(Intercept)	114.70	91.41	2.03		
	GDD0.s1	−0.10	−6.41	0.211	−30.44	top +4
	GDD12.m3	−0.10	1.16	0.19	6.21	top +1
	GDD10.m5	1.31	−1.20	0.21	−5.70	top +1
BEGIN FLOWERING Late	(Intercept)	74.64	114.68	1.83		
	Begin Bud Burst	0.42	4.04	0.16	24.76	top +1
	GDD8.s1	−0.07	−3.46	0.36	−9.55	top +4
	CDD8.m2	0.02	1.11	0.37	2.99	top +1
BEGIN LEAF COLOR OB sapling	(Intercept)	208.77	195.79	2.31		
	Rain days.m4	1.69	3.77	0.70	5.42	top +4
	Mean temp.s2	−1.84	−2.80	0.70	−4.02	top +3
	Hot days.s1	−0.42	−2.58	0.70	−3.68	top +1
BEGIN LEAF COLOR OB tree	(Intercept)	284.38	244.34	1.99		
	Mean.t.s1	−2.03	−2.38	0.48	−4.99	top +3
	Drought.m4	3.14	2.08	0.47	4.44	top +4
	Rain.s1	0.52	1.82	0.47	3.85	top +1
BEGIN LEAF COLOR Intermediate	(Intercept)	266.77	276.93	1.39		
	Min.t.m1	1.49	2.49	0.16	15.97	top +4
	Drought.m3	−2.29	−2.01	0.25	−8.03	top +0
	Precip.s2	−0.02	−2.18	0.41	−5.36	top +2
BEGIN LEAF COLOR Late	(Intercept)	261.74	292.03	1.13		
	Min.t.s1	3.42	4.37	0.22	19.53	top +4
	Drought.m3	−1.63	−1.39	0.12	−11.63	top +0
	Min.t.m6	−1.07	−1.70	0.20	−8.32	top +4
BEGIN LEAF DROP OB sapling	(Intercept)	11.40	209.27	0.63		
	Begin Leaf Color	1.02	14.75	0.53	27.68	top +4
	Hot.m1	−0.38	−2.30	0.57	−4.05	top +3
	Mean.t.s3	0.68	1.45	0.56	2.60	top +0
BEGIN LEAF DROP OB tree	(Intercept)	69.17	255.86	0.46		
	Begin Leaf Color	0.90	11.32	0.34	33.60	top +4
	Mean.t.m4	−1.80	−2.58	0.30	−8.55	top +4
	Rain.m2	0.66	1.61	0.30	5.39	top +0
BEGIN LEAF DROP Intermediate	(Intercept)	75.67	291.31	0.68		
	Begin Leaf Color	0.75	8.06	0.38	21.29	top +4
	Min.t.s1	0.85	1.07	0.22	4.87	top +4
	Min.t.m6	−0.42	−0.67	0.18	−3.79	top +0
BEGIN LEAF DROP Late	(Intercept)	95.52	305.69	0.88		
	Begin Leaf Color	0.55	7.04	0.50	14.21	top +4
	Min.t.m6	−0.70	−1.02	0.12	−8.49	top +2
	Mean.t.m2	0.71	1.04	0.22	4.61	top +0

*Note:* See Appendix A: Table A6 for species within each group. Analyses were conducted on standardized values for explanatory variables because the variables were on different scales. Model coefficients (slope or intercept), standard errors, and *t*‐values are reported from models that use standardized values. However, the coefficients from models created using the raw values of explanatory variables are also given for reference (“raw coefficient” column). Also shown are the number of additional occurrences of each variable in the next four top models. See Appendix C: Tables C5a and C5b for the next four top models.

**TABLE C7a:** Species‐level estimated coefficients for the slope of each spring phenological event's response to the most influential weather variable. The most influential weather variable was determined as the one with the greatest absolute t‐value in the top model, and that was also present in at least four of the top five models (see Appendix C: Tables C5a, C5b, and C6).

Spring events	Begin Bud Swell—OB Sap	Begin Bud Swell—Late	Begin Bud Burst—OB Sap	Begin Bud Burst—Late	Begin Leaf Expansion—OB Sap + Tree	Begin Leaf Expansion—Late	End Leaf Expansion—OB Sap + Tree	End Leaf Expansion—Intermediate	End Leaf Expansion—Late	Begin Flowering—Early	Begin Flowering—Late
Weather variable	GDD2.m1	GDD2.m1	GDD0.m1	GDD4.m1	GDD10.s1	GDD8.s1	GDD2.s1	GDD6.s1	GDD2.s1	GDD0.s1	GDD8.s1
Species											
*Tilia americana* Amer. basswood canopy tree		−0.054		−0.073		−0.033		−0.020			NA
*Ulmus americana* American elm canopy tree		−0.078		−0.046		−0.033		−0.022		−0.097	
*Carya cordiformis* bitternut hickory canopy tree		−0.074		−0.065		−0.034		−0.021			−0.070
*Juglans nigra* black walnut canopy tree		−0.088		−0.059		−0.027			−0.029		−0.061
*Fraxinus quadrangulata* blue ash canopy tree		−0.070		−0.064		−0.035		−0.023			−0.068
*Quercus macrocarpa* bur oak canopy tree		−0.103		−0.089		−0.035		−0.021			−0.101
*Celtis occidentalis* common hackberry canopy tree		−0.115		−0.105		−0.046		−0.022			−0.008
*Fraxinuus pennsylvanica* green ash canopy tree		−0.069		−0.053		−0.035		−0.022			−0.070
*Gymnocladus dioicus* Kentucky coffeetree canopy tree		−0.082		−0.051		−0.030			−0.025		NA
*Quercus rubra* northern red oak canopy tree		−0.096		−0.083		−0.037		−0.026			−0.102
*Aesculus glabra* Ohio buckeye canopy tree		−0.087		−0.062	−0.082		−0.059				−0.096
*Carya laciniosa* shellbark hickory canopy tree		−0.072		−0.073		−0.027		−0.020			−0.073
*Ulmus rubra* slippery elm canopy tree		−0.054		−0.051		−0.032		−0.013		−0.097	
*Acer saccharum* sugar maple canopy tree		−0.053		−0.063		−0.032		−0.021			−0.060
*Fraxinus americana* white ash canopy tree		−0.061		−0.050		−0.029		−0.020			−0.074
*Carpinus caroliniana* Amer. hornbeam treelet		−0.086		−0.069		−0.036		−0.022			−0.065
*Asimina triloba* pawpaw treelet		−0.086		−0.113		−0.039			−0.052		−0.066
*Zanthoxylum americanum* common pricklyash shrub		−0.076		−0.074		−0.036			−0.037		−0.082
*Lindera benzoin* northern spicebush shrub		−0.049		−0.045		−0.047		−0.020		−0.097	
*Toxicodendron radicans* eastern poison ivy vine		−0.063		−0.078		−0.031		−0.026			NA
*Vitis* spp. grape species mix vine		−0.079		−0.055		−0.033		N/A			NA
*Parthenocissus quinquefolia* Virginia creeper vine		−0.088		−0.074		−0.034		−0.027			NA
*Fraxinus quadrangulata* blue ash sapling		−0.054		−0.050		−0.035		−0.025			NA
*Aesculus glabra* Ohio buckeye sapling	−0.122		−0.069		−0.082		−0.059				NA
*Acer saccharum* sugar maple sapling		−0.076		−0.046		−0.035		−0.023			NA

*Note:* Only species with sufficient data for analysis are included. The slope represents the thermal sensitivity, that is, change (number of days) in the phenological event date in response to one unit of the weather variable: that is, the slope is *b* in the equation: Event date = *b* (weather variable) + intercept. All slopes are based on raw values of weather variables—that is, untransformed values.

**TABLE C7b:** Species‐level estimated coefficients for the thermal sensitivity, that is, slope of each autumn phenological event's response to the most influential weather variable.

Autumn events	Begin Leaf Coloration—OB sap	Begin Leaf Coloration—OB Tree	Begin Leaf Coloration—Intermediate	Begin Leaf Coloration—Late	Begin Leaf Drop—OB sap	Begin Leaf Drop—OB tree	Begin Leaf Drop—Intermediate	Begin Leaf Drop—Late
Weather Variable	Rain.m4	Mean.T.s1	Min.T.m1	Min.T.s1	Hot.m1	Mean.T.m4	Min.T.s1	Min.T.m6
Species								
*Tilia americana* Amer. basswood canopy tree			1.488				1.008	
*Ulmus americana* American elm canopy tree				3.767				−0.581
*Carya cordiformis* Bitternut hickory canopy tree				3.658				−0.725
*Juglans nigra* Black walnut canopy tree			1.488				−0.144	
*Fraxinus quadrangulata* Blue ash canopy tree								−0.755
*Quercus macrocarpa* Bur oak canopy tree				3.598				−0.713
*Celtis occidentalis* Common hackberry canopy tree				2.564				−0.731
*Fraxinuus pennsylvanica* Green ash canopy tree			1.488				0.785	
*Gymnocladus dioicus* Kentucky coffeetree canopy tree			1.488				0.468	
*Quercus rubra* Northern red oak canopy tree				3.727				−0.550
*Aesculus glabra* Ohio buckeye canopy tree		−2.028				−1.805		
*Carya laciniosa* Shellbark hickory canopy tree				3.500			1.150	
*Ulmus rubra* Slippery elm canopy tree				3.375			0.698	
*Acer saccharum* Sugar maple canopy tree				3.252				−0.880
*Fraxinus americana* White ash canopy tree			1.488				0.685	
*Carpinus caroliniana* Amer. hornbeam treelet				3.884			0.559	
*Asimina triloba* Pawpaw treelet				3.692				−0.712
*Zanthoxylum americanum* Common pricklyash shrub				2.951				−0.663
*Lindera benzoin* Northern spicebush shrub				3.270			0.923	
*Toxicodendron radicans* Eastern poison ivy vine			1.488				1.22	
*Vitis* spp. grape specie mix vine			1.488				1.666	
*Parthenocissus quinquefolia* Virginia creeper vine			1.488				1.136	
*Fraxinus quadrangulata* Blue ash sapling				3.301				−0.549
*Aesculus glabra* Ohio buckeye sapling	1.686				−0.378			
*Acer saccharum* Sugar maple sapling				3.130				−0.767

*Note:* See Appendix C: Table C7a for details.

**TABLE C8:** For each phenological event, a summary of the seasonal group‐level thermal sensitivity, that is, shift (number of days) per unit change in the top weather variable.

Event	Seasonal group	Weather variable	Days change/Weather unit change	No. Species
Begin Bud Swell	OB sapling	GDD2.m1	0.11 days advance/GDD	1
	Late	GDD2.m1	0.08 days advance/GDD	24
Begin Bud Burst	OB sapling	GDD0.m1	0.07 days advance/GDD	1
	Late	GDD4.m1^a^	0.07 days advance/GDD	24
Begin Leaf Expansion	OB sapling + tree	GDD10.s1^b^	0.08 days advance/GDD	2
	Late	GDD8.s2^b^	0.03 days advance/GDD	23
End Leaf Expansion	OB sapling + tree	GDD2.s1	0.06 days advance/GDD	2
	Intermediate	GDD6.s1^c^	0.02 days advance/GDD	18
	Late	GDD2.s1^c^	0.04 days advance/GDD	4
Begin Flowering	Early	GDD0.s1	0.10 days advance/GDD	3
	Late	GDD8.s1^b^	0.07 days advance/GDD	14
Begin Leaf Coloration	OB sapling	Rain days.m4	1.69 days delay/Rain da	1
	OB tree	Mean T.s1	2.03 days advance/° (C) Mean t	1
	Intermediate	Min T.m1	1.49 days delay/Min t	8
	Late	Min T.s1	3.42 days delay/Min t	15
Begin Leaf Drop	OB sapling	Hot days.m1^d^	0.38 days advance/Hot da	1
	OB tree	Mean T.m4^d^	1.80 days advance/Mean t	1
	Intermediate	Min T.s1^d^	0.85 days delay/Min t	11
	Late	Min T.m6^d^	0.70 days advance/Min t	11

*Note:* The most influential weather variable had the greatest absolute *t*‐value in the top model, and was also present in at least four of the top five models (see Appendix C: Tables C5a and C5b). All species within a group show the same direction of change with an increase in the weather variable (negative (advance) or positive (delay)), except 1 of 12 species for Begin Leaf Drop (Intermediate). Also shown are number of species in each group. See Appendix A: Table A5 for species in each group.

^a^ The strongest weather predictor, but Date of Begin Bud Swell performed stronger as phenological explanatory variable.

^b^ The strongest weather predictor, but Date of Begin Bud Burst performed stronger as phenological explanatory variable.

^c^ The strongest weather predictor, but Date of Begin Leaf Expansion performed stronger as phenological explanatory variable.

^d^ The strongest weather predictor, but Date of Begin Leaf Coloration performed stronger as phenological explanatory variable.

**TABLE C9a:** Results of regression analysis for key weather predictors of spring events as a function of year (1993–2021). The phenological event and seasonal group for which the weather variab was important is given in the leftmost column.

Event—Seasonal Group	Response	Statistic	Estimate	Std. Error	*t*	*p*
BEGIN BUD SWELL—Ohio Buckeye Sapling	GDD_2_, DOY 51–80	Intercept	108.0	20.0		
		Slope	1.18	1.22	0.962	0.345
		*R* ^2^	0.033			
	CDD_4_, DOY 356–20	Intercept	248.6	31.0		
		Slope	−2.39	1.90	−1.260	0.218
		*R* ^2^	0.056			
BEGIN BUD SWELL—Late Species	GDD_2_, DOY 70–99	Intercept	150.9	24.1		
		Slope	1.42	1.48	0.962	0.345
		*R* ^2^	0.020			
BEGIN BUD BURST—Ohio Buckeye Sapling	GDD_0_, DOY 61–90	Intercept	149.1	24.8		
		Slope	1.49	1.52	0.980	0.336
		*R* ^2^	0.021			
BEGIN BUD BURST—Late Species	GDD_4_, DOY 80–109	Intercept	156.2	18.7		
		Slope	1.13	1.14	0.986	0.333
		*R* ^2^	0.035			
BEGIN LEAF EXPANSION—Ohio Buckeye Sapling + Tree	GDD_10_, DOY 44–103	Intercept	39.2	12.1		
		Slope	0.86	0.74	1.160	0.256
		*R* ^2^	0.047			
BEGIN LEAF EXPANSION—Late Species	GDD_8_, DOY 66–125	Intercept	169.6	22.3		
		Slope	1.42	1.37	1.036	0.309
		*R* ^2^	0.038			
END LEAF EXPANSION—Ohio Buckeye Sapling + Tree	GDD_2_, DOY 56–115	Intercept	332.1	29.9		
		Slope	1.76	1.83	0.958	0.347
		*R* ^2^	0.033			
END LEAF EXPANSION—Intermediate Species	GDD_6_, DOY 75–134	Intercept	336.0	28.3		
		Slope	0.65	1.74	0.373	0.712
		*R* ^2^	0.005			
END LEAF EXPANSION—Late Species	GDD_2_, DOY 94–153	Intercept	725.6	25.5		
		Slope	2.57	1.57	1.642	0.112
		*R* ^2^	0.091			
BEGIN FLOWERING—Early Species	GDD_0_, DOY 32–91	Intercept	219.2	31.0		
		Slope	1.04	1.97	0.527	0.602
		*R* ^2^	0.011			
BEGIN FLOWERING—Late Species	GDD_8_, DOY 56–115	Intercept	119.2	18.8		
		Slope	1.04	1.15	0.902	0.375
		*R* ^2^	0.029			

*Note:* In all cases, the linear regression was conducted such that the weather variable was the response, and the year was the explanatory variable where *year* = 0 for 1993 (the first year of the study). Also, in all cases the *p*‐value was calculated from a *t*‐distribution with df = 27. Weather variables analyzed through time were selected based on their consistency in appearing in at least four of the top five models (see Appendix C: Tables C5a and C5b) rather than in only the single top model (Appendix C: Table C6). Some events have multiple important weather variables.

**TABLE C9b:** Results of regression analysis for key weather predictors of autumn events as a function of year (1993–2021).

Event—Seasonal Group	Response	Statistic	Estimate	SE	*t*	*p*
BEGIN LEAF COLORATION—Ohio Buckeye Sapling	Rain days, DOY 75–104	Intercept	6.3	0.9		
		Slope	0.04	0.05	0.733	0.470
		*R* ^2^	0.020			
	Mean daily mean temp, DOY 135–194	Intercept	10.9	0.6		
		Slope	0.01	0.03	0.382	0.706
		*R* ^2^	0.005			
BEGIN LEAF COLORATION—Ohio Buckeye Tree	Mean daily mean temp, DOY 184–243	Intercept	23.5	0.4		
		Slope	0.01	0.03	0.249	0.805
		*R* ^2^	0.002			
	Drought weeks, DOY 124–153	Intercept	0.6	0.2		
		Slope	−0.01	0.02	−0.882	0.386
		*R* ^2^	0.028			
BEGIN LEAF COLORATION—Intermediate Species	Mean daily minimum temp, DOY 246–275	Intercept	11.3	0.6		
		Slope	0.11	0.03	3.201	0.003
		*R* ^2^	0.275			
BEGIN LEAF COLORATION—Late Species	Mean daily minimum temp, DOY 232–291	Intercept	11.4	0.4		
		Slope	0.08	0.03	2.828	0.009
		*R* ^2^	0.229			
	Mean daily minimum temp, DOY 112–141	Intercept	8.9	0.6		
		Slope	0.01	0.35	0.214	0.832
		*R* ^2^	0.002			
BEGIN LEAF DROP—Ohio Buckeye Sapling	Number of hot days, DOY 179–208	Intercept	6.7	2.3		
		Slope	0.05	0.14	0.323	0.749
		*R* ^2^	0.004			
BEGIN LEAF DROP—Ohio Buckeye Tree	Mean daily mean temp, DOY 136–165	Intercept	18.2	0.4		
		Slope	0.11	0.03	4.565	< 0.0001
		*R* ^2^	0.436			
BEGIN LEAF DROP—Intermediate Species	Mean daily minimum temp, DOY 232–291	Intercept	11.4	0.4		
		Slope	0.08	0.03	2.828	0.009
		*R* ^2^	0.229			
BEGIN LEAF DROP—Late Species	No weather variables were in four of the top five models.					

*Note:* See Appendix C: Table C9a for details.

**TABLE C10a:** Standard deviation of random intercepts (for individuals and species‐growth form) and random species slopes (for fixed effects) from the top linear mixed‐effects models for weather variables predicting spring phenological events.

Event	Random effects	SD
Seasonal group		
BEGIN BUD SWELL Early Ohio Buckeye sapling	Intercept—individual	0
	(residual)	5.00
BEGIN BUD SWELL Late species	Intercept—individual	1.65
	Intercept—species‐form	5.10
	CDD_8_.s1	1.12
	GDD_2_.m1	0.88
	CDD_4_.s2	0.45
	(residual)	4.99
BEGIN BUD BURST Early Ohio Buckeye sapling	Intercept—individual	1.18
	(residual)	3.60
BEGIN BUD BURST Late species	Intercept—individual	1.12
	Intercept—species‐form	2.37
	Begin Bud Swell	1.05
	GDD_4_.m1	0.93
	Precip.Previous Autumn	0.56
	(residual)	3.80
BEGIN LEAF EXPANSION Early OB Sapling + Tree	Intercept—individual	0.22
	Intercept—species‐form	0.98
	(residual)	2.83
BEGIN LEAF EXPANSION Late species	Intercept—individual	0.67
	Intercept—species‐form	2.62
	Precip.PreviousAutumn	0.52
	GDD_8_.s1	0.60
	Begin Bud Burst	0.65
	(residual)	3.59
END LEAF EXPANSION Early OB Sapling + Tree	Intercept—individual	0.81
	Intercept—species‐form	1.69
	(residual)	5.12
END LEAF EXPANSION Intermediate species	Intercept—individual	0.53
	Intercept—species‐form	2.18
	Flood.m3	0.98
	GDD_6_.s1	0.64
	Begin Leaf Expansion	0.61
	(residual)	4.53
END LEAF EXPANSION Late species	Intercept—individual	1.99
	Intercept—species‐form	5.93
	Begin Leaf Expansion	1.58
	GDD_2_.s1	1.29
	(residual)	6.47
BEGIN FLOWERING Early species	Intercept—individual	0
	Intercept—species‐form	3.51
	(residual)	4.39
BEGIN FLOWERING Late species	Intercept—individual	1.11
	Intercept—species‐form	6.83
	CDD8.m2	1.30
	GDD8.s1	1.21
	(residual)	4.40

*Note:* The top model could include up to three fixed effects with random slopes. Fixed effects included weather variables or legacy events that preceded the event in question. Analyses were conducted for each of five phenological events, each separated into seasonal groups (a total of 11 event–group combinations). Standard deviation indicates variability in the random effect (intercept or slope). These calculations are based on models constructed with standardized fixed effects (transformed to have a mean of zero and standard deviation of one). See Appendix A: Table A5 for species in each seasonal group. See Appendix A: Tables A6, A7a, and A7b for descriptions of abbreviated weather variables.

**TABLE C10b:** Standard deviation of random intercepts (for individuals and species‐growth form) and random species slopes (for fixed effects) from the top linear mixed‐effects models for weather variables predicting autumn phenological events. Analyses were conducted for each of two autumn phenological events, each separated into seasonal groups (a total of 8 event–group combinations).

Event	Random effects	SD
Seasonal group		
BEGIN LEAF COLORATION Early Ohio Buckeye Sapling	Intercept—individual	7.00
	(residual)	11.43
BEGIN LEAF COLORATION Early Ohio Buckeye Tree	Intercept—individual	7.90
	(residual)	9.54
BEGIN LEAF COLORATION Intermediate species	Intercept—individual	3.75
	Intercept—species‐form	3.73
	Drought.m3	1.01
	Precip.s2	0.47
	(residual)	7.38
BEGIN LEAF COLORATION Late species	Intercept—individual	3.42
	Intercept—species‐form	4.18
	Min.t.s1	0.74
	Min.t.m6	0.66
	Drought.m3	0.22
	(residual)	6.45
BEGIN LEAF DROP Early Ohio Buckeye Sapling	Intercept—individual	1.25
	(residual)	8.25
BEGIN LEAF DROP Early Ohio Buckeye Tree	Intercept—individual	1.44
	(residual)	6.10
BEGIN LEAF DROP Intermediate species	Intercept—individual	1.54
	Intercept—species‐form	2.26
	Begin Leaf Color	1.19
	Min.t.s1	0.66
	Min.t.m6	0.52
	(residual)	5.11
BEGIN LEAF DROP Late species	Intercept—individual	2.25
	Intercept—species‐form	2.73
	Begin Leaf Coloration	1.52
	Mean.t.m2	0.58
	Min.t.m6	0.19
	(residual)	5.11

*Note:* See Appendix C: Table C10a for other details.
